# Synthesis of the heterocyclic core of the D-series GE2270

**DOI:** 10.3762/bjoc.13.137

**Published:** 2017-07-17

**Authors:** Christophe Berini, Thibaut Martin, Pierrik Lassalas, Francis Marsais, Christine Baudequin, Christophe Hoarau

**Affiliations:** 1 Normandie Univ, INSA Rouen, UNIROUEN, CNRS, COBRA (UMR6014), 76000 Rouen (France), Rue Tesnière, 76821 Mont-Saint-Aignan Cedex, France

**Keywords:** antibiotic, bromination, BSC, C–H arylation, cross-coupling, Hantzsch synthesis, thiopeptide

## Abstract

A straightforward enantiomerically pure synthesis of the heterocyclic core of the D-series GE2270 is reported. The synthetic strategy combines the Hantzsch thiazole’s building condensation with a cross-coupling reaction including direct C–H hetarylation to build and connect step-by-step thiazolyl moieties to the 5-bromopicolinate as readily available starting material.

## Introduction

**Figure 1:**
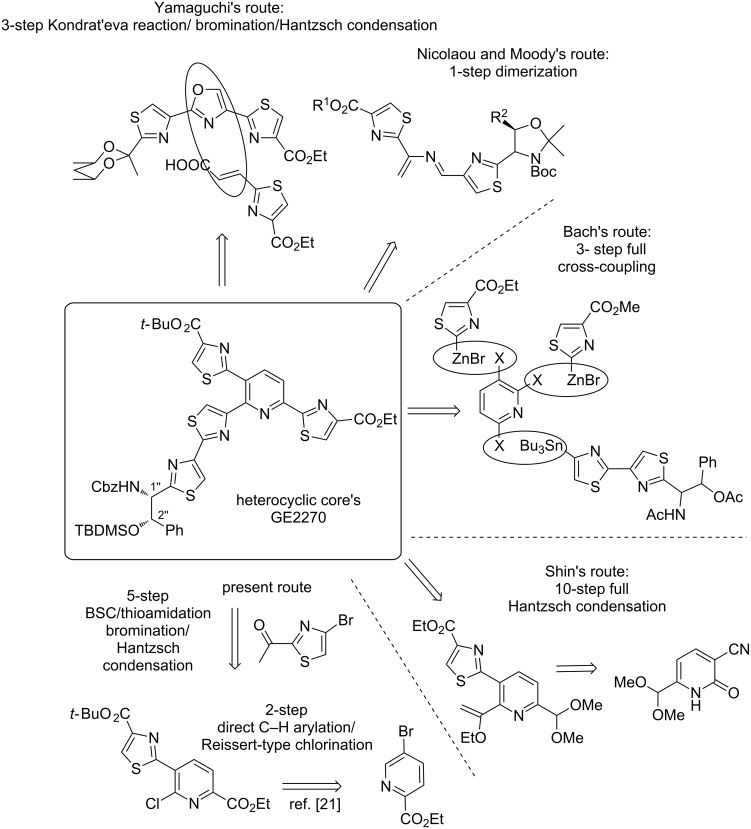
Main synthetic strategies towards heterocyclic cores of D-series GE2270 and our present one.

Thiopeptide antibiotics are a class of peptide-derived macrocycles which contain many thiazole and thiazoline units, with almost 90 structures organized into 32 families and 5 series [1–9]. The particularity of these molecules is the interruption of the modified cyclopeptide chain by a more complex nitrogen-containing heterocycle found at different oxidation states (from series *a* to *e*) and bearing many azole units. They inhibit bacterial protein synthesis through two main modes of action, most of them bind the complex of 23S rRNA with ribosomal protein L11 and few of them such as the thiopeptide of D-series GE2270 modify the action of elongation factors Tu. The most pharmalogically-advanced thiopeptide antibody of D-series LFF571 developed by Novartis is currently evaluated for treating *Clostridium difficile* intestinal infections [10]. Due to their important biological activity, many groups such as Moody, Bagley, Bach, Nicolaou, Hashimoto–Nakata, Ciufolini and Alvarez are actively involved in the total synthesis of these architecturally-sophisticated macrolides [11]. One of multiple synthetic challenges is the development of concise synthetic routes toward the complex heterocyclic cores. To date, two main synthetic general strategies towards the most represented di- or trithiazolylpyridine heterocyclic cores of the D-series thiopeptides have been explored, (i) the late-stage construction of the pyridine core through [4 + 2] cycloaddition reaction flanked with the main difficulties to prepare the dienophile and alkene bearing the adequate thiazolyl moieties or (ii) the direct connection and construction of thiazole units using cross-coupling reactions and Hantzsch-type condensation to a pyridine central platform. This second strategy has been first initially explored by Kelly in 1991 for the first preparation of heterocyclic core of micrococcinic acid [12–13] but due to the unique and original mode of bacterial protein synthesis of GE2270, the preparation of the common tri-thiazolylpyridine heterocyclic core of this family has driven many attention [11]. Shin first prepared the GE2270 heterocyclic core in 9% yield over a 10-step sequence (Figure 1) starting with a highly pre-functionalized pyridinone within five Hantzsch thiazole building steps [14]. Nicolaou and Moody reported then the synthesis of the heterocyclic core of GE2270 by using a late-stage [4 + 2] cycloaddition of a sophisticated bithiazolylazadiene with alkynylated thiazole [15–16]. As highly innovative strategy, Bach reported in 2005 the neat synthesis of GE2270 central core in 33% yield over a 4-step sequence (Figure 1) including three Negishi and Stille cross-coupling reactions to achieve the direct introduction of mono- and dithiazolyl units to a 2,3,6-trihalopyridine [17]. The strategy has then extended to the total synthesis of thiopeptides GE2270 in which the macrocylization and heterocyclic core was simultaneously achieved through a late-stage Negishi cross-coupling [18–19]. Last year, Yamaguchi’s group proposed a novel elegant [4 + 2] cycloaddition Kondrat’eva reaction between a 2-alkenylated thiazole-4-carboxylate with a sophisticated tris-1,3-diazole, judiciously prepared through direct C–H arylation method, to synthesize the trithiazolylpyridine intermediate (Figure 1). The latter was then transformed to heterocycle core of GE2270 by adding a final bromination/Hantzsch sequence to build the last 2,4’-bithiazole appendage [20]. In 2009, we have reported an original access to micrococcinate ester (R = Et) from ready available ethyl 5-bromo picolinate (Figure 1) [21–22]. The synthetic strategy was based upon the step-by-step direct introduction and building of thiazolyl units onto a pyridine central core by using two developed innovative cross-coupling reactions, the palladium-catalyzed direct C–H (hetero)arylation of thiazole-4-carboxylate [21] and a palladium-catalyzed borylation-Suzuki coupling (BSC) 2-ketothiazole unit at 4-position as alternative to thiazolyltin intermediate [22]. Herein, an improved synthetic strategy toward the structurally-related micrococcinate ester bearing a methyl ketone function (R = Me) was reported through building and direct introduction of thiazole units using BSC and Hantzsch methodologies. The final access to a novel fully orthogonally-protected heterocyclic core of GE2270 was achieved by adding a final bromination/Hantzsch thiazole synthesis to build the adequate 2,4’-bithiazole appendage.

## Results and Discussion

Our first investigations were directed towards the 6-chloro-5-thiazolylpicolinate ester **4** in multi-gram amounts. We found that our previously reported protocol of direct C–H arylation of *tert*-butyl thiazole-4-carboxylate (**2**) with 5-bromopicolinate ester **1** [21] could be scaled-up from milligram to multigram scale. The subsequent access to the 6-chloro-5-thiazolylpicolinate ester **4** was then achieved in good yields by following our previously reported two-step oxidation/Reissert-type sequence [21–22]. The access to the key intermediate trithiazolylpyridine **9** was next investigated via two synthetic routes by exploiting BSC cross-coupling and Hantzsch condensation reactions from the picolinamide **5**, which was produced by simple treatment of 6-chloro-5-thiazolylpicolinate ester **4** with ammonia in excellent 93% yield. Our previous synthetic route towards micrococcinate ester as structurally-related analog was first envisaged. The first route consists first in a two-step Hantzsch-type thiazole construction to produce the 6-chlorobithiazolylpyridine **7** in fair 68% yield over 2 steps. Next, the strict use of our previous protocol of BSC cross-coupling of methyl(4-bromothiazol-2-yl)ketone (**8**) with 6-chlorobithiazolylpyridine **7** [22] that employs bis(pinacolato)diboron (B_2_pin_2_) as borylating agent and Pd_2_(dba)_3_/Cy-JohnPhos pair as optimal catalyst led to the fair production of the expected trithiazolylpyridine **9** but in moderate 46% yield. Nevertheless, an additional screening of palladium sources has revealed that Pd(OAc)_2_ was a better pre-catalyst to perform quantitatively the first borylation-step in short reaction time (1 h) and to achieve subsequently the cross-coupling with methyl(4-bromothiazol-2-yl)ketone (**8**) providing the excepted trithiazolylpyridine **9** in an excellent 82% yield.

**Scheme 1:**
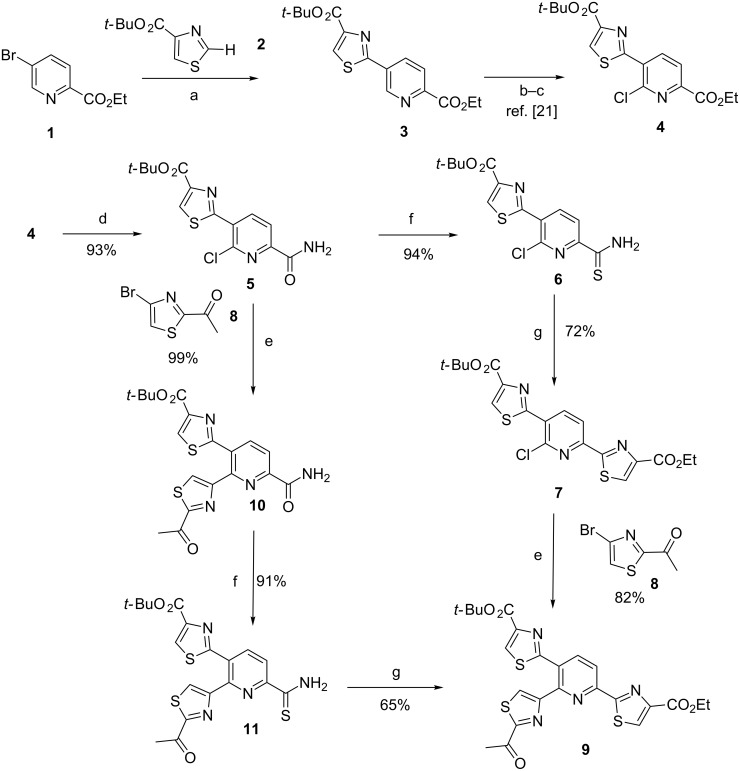
Synthesis of trithiazolylpyridine **9**. Reaction conditions: a) Pd(OAc)_2_ (5 mol %), CyJohnPhos (10 mol %), Cs_2_CO_3_ (2 equiv), DMF, 110 °C, 18 h, 69%. b) UHP (2 equiv), TFAA (2.1 equiv), ACN, 0 °C, 1 h, quantitative. c) POCl_3_ (2 equiv), toluene/DMF, 0 °C, 1 h, 83%. d) NH_4_OH, THF/H_2_O (2:1), rt, 14 h, 93%. e) (i) **8**, Bis(pinacolato)diboron, Pd(OAc)_2_ (5 mol %), CyJohnPhos (20 mol %), KOAc, dioxane, 1 h; (ii) K_3_PO_4_, dioxane/H_2_O (4:1), 110 °C, 14 h, 82–99%. f) Lawesson’s reagent, CH_2_Cl_2_, 40 °C, 12 h, 91–94%. g) Ethyl bromopyruvate, CaCO_3_, THF/EtOH (1:1), 60 °C, 24 h.

At this stage, we set out to evaluate a second innovative synthetic route towards the trithiazolylpyridine **9** through a reverse Hantzsch condensation/BSC reactions sequence to build and introduce both thiazole units to the picolinamide **5** (Scheme 1).

Pleasingly, the application of the optimized BSC procedure based upon the use of Pd(OAc)_2_ as pre-catalyst allowed to achieve the cross-coupling of picolinamide **5** with methyl(4-bromothiazol-2-yl)ketone (**8**), providing the expected bithiazolylpicolinamide **10** in quantitative yield. Remarkably, no side Buchwald–Harwig heterarylation of the amide function was observed. Next, following the same two-step Hantzsch thiazole building protocol employed within the first synthetic route, the expected trithiazolylpyridine **9** was isolated in fair 59% yield over 2 steps. Finally, the second synthetic pathway proved to be slightly more performant to produce the trithiazolylpyridine key-intermediate **9** from the picolinamide **5** in 58% yields over 3 steps (vs 55% for the first synthetic route).

**Scheme 2:**
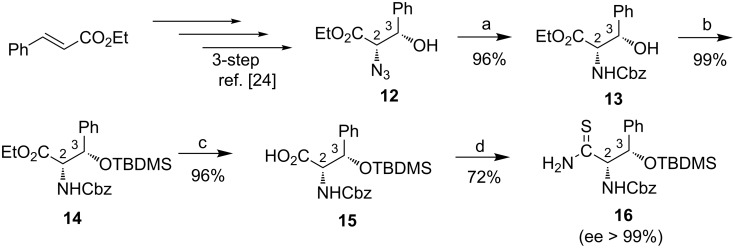
Synthesis of chiral thioamide **16**. Reaction conditions: a) SnCl_2_∙2H_2_O, dioxane/H_2_O (1:3), 0 °C to rt, 5 h, then NaHCO_3_, benzyl chloroformate, rt, 18 h, 96%. b) TBDMSCl, imidazole, DMF, rt, 16 h, 99%. c) LiOH∙H_2_O, DME/H_2_O (1:1), 0 °C to rt, 72 h, then HCl, 96%. d) DCC, HOSu, THF, rt, 16 h, then Lawesson’s reagent, DME, rt, 36 h, 72%.

Then, the trithiazolylpyridine **9** was engaged in an additional bromination reaction followed by Bagley’s modified Hantzsch condensation with the adequate thioamide to deliver the fully orthogonally-protected heterocyclic core of GE2270 along with avoiding racemization of stereogenic centers (Figure 1). Successfully applied last year by Yamagushi's group from a structurally analog of **9** in DCM as the solvent [20], the Pattenden two-step enol silylation/bromination sequence [23] also found effective to produce consecutively the *tert*-butyldimethylsilyl enol-ether intermediate from trithiazolylpyridine **9**. The latter was treated with NBS reagent in THF to give the expected brominated trithiazolylpyridine **17** in 51% yield over two steps. The enantiomerically pure Cbz-protected thioamide **16** was newly synthesized from the commercially available ethyl *trans*-cinnamate in 7 steps and 27% overall yield following the Nicolaou reported synthetic pathway [24] (Scheme 2).

**Scheme 3:**
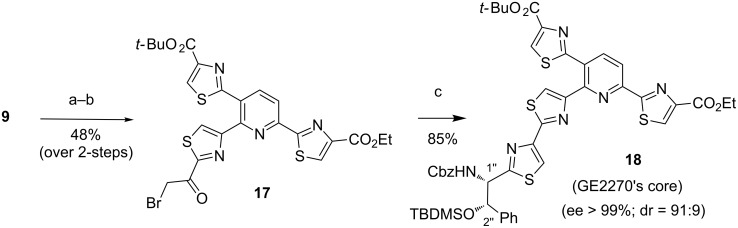
Synthesis of the heterocyclic core of the D-series GE2270. Reaction conditions: a) TBDMSOTf, NEt_3_, DCM, 0 °C, 1 h, 51%. b) NBS, THF, 1 h, 94%. c) **16**, MS 4 Å, DMF, 0 °C, 14 h, then 2,6-lutidine, TFAA, DME, −20 °C, 12 h, 85%.

As final stage, the Hantzsch condensation of brominated trithiazolylpyridine **17** with *N*-Cbz/*O*-TBDMS-bis-protected thioamide **16** was investigated, inspired by the modified Merritt and Bagley protocol, which was specifically designed to avoid a strong acidification of the reaction and thus prevent side-hydrolysis of sensitive functions, as well as epimerization of stereocenters [25]. The first condensation step leading to the thiazoline intermediate was successfully achieved, although in the presence of molecular sieves without KHCO_3_. The latter was then immediately dehydrated at low temperature using the recommended TFAA reagent providing the expected heterocyclic core of D-series GE2270 **18** with a high diastereoisomeric ratio (91:9) and high enantiomeric purity (>99%) in 41% yield over 3 steps (Scheme 3).

## Conclusion

In summary, the fully orthogonally protected and enantiomerically pure heterocyclic core of the D-series thiopeptide antibiotic GE2270 was prepared. The synthetic strategy that combines direct C–H arylation, Borylation Suzuki–Miyaura cross-coupling (BSC) and Hantzsch thiazole synthesis methods proved to be highly effective regarding the fair 22% yield over 7 synthetic steps from the key-intermediate 6-chloro-5-thiazolylpicolinate ester **4**, which was obtained in multi-gram amounts in 57% yield over 3 synthetic steps (13% global yield) from the readily available ester ethyl 5-bromopicolinate (**1**) as starting material.

## Supporting Information

File 1 Experimental procedures and NMR spectra.
