# Supplementary material for: Synthesis of the heterocyclic core of the D-series GE2270
Source: Beilstein J Org Chem. 2017 Jul 17;13:1407–12. doi: 10.3762/bjoc.13.137 (PMC5530635; doi:10.3762/bjoc.13.137)
Supplement: File 1 — Experimental procedures and NMR spectra. [file Beilstein_J_Org_Chem-13-1407-s001.pdf]

Supporting Information

for

Synthesis of the heterocyclic core of the D-series GE2270

Christophe Berini[1], Thibaut Martin[1], Pierrik Lassalas[1], Francis Marsais[1], Christine Baudequin[1] and Christophe Hoarau*[1]

Address: [1]Normandie Univ,INSA Rouen, UNIROUEN, CNRS, COBRA (UMR6014), 76000 Rouen (France), 1 Rue Tesnière 76821 Mont-Saint-Aignan Cedex, France.

Email: Christophe Hoarau - Christophe.hoarau@insa-rouen.fr

*Corresponding author

Experimental procedures and NMR spectra.

Table of Contents

Table of Contents S2

I. General Information S4

II. Experimental Procedures S6

1. Synthesis of the trithiazolylpyridine 9 .... S6

a) *tert*-Butyl 2-(2-(2-acetylthiazol-4-yl)-6-carbamoylpyridin-3-yl)thiazole-4-carboxylate (**10**). S6

b) *tert*-Butyl 2-(2-(2-acetylthiazol-4-yl)-6-carbamothioylpyridin-3-yl)thiazole-4-carboxylate (**11**). S7

c) *tert*-Butyl 2-(2-chloro-6-(4-(ethoxycarbonyl)thiazol-2-yl)pyridine-3-yl)thiazole-4-carboxylate (**7**). S8

d) *tert*-Butyl 2-(2-(2-acetylthiazol-4-yl)-6-(4-(ethoxycarbonyl)thiazol-2-yl)pyridin-3-yl)thiazole-4-carboxylate (**9**). S9

2. Synthesis of chiral thioamide 16 .... S11

a) (2*S*,3*S*)-Ethyl-2-(benzyloxycarbonylamino)-3-hydroxy-3-phenylpropanoate (**13**). S11

b) (2*S*,3*S*)-Ethyl 2-(benzyloxycarbonylamino)-3-(*tert*-butyldimethylsilyloxy)-3-phenylpropanoate (**14**). S12

c) (2*S*,3*S*)-2-(Benzyloxycarbonylamino)-3-(*tert*-butyldimethylsilyloxy)-3-phenylpropanoic acid (**15**). S13

d) Benzyl (2*S*,3*S*)-1-amino-3-(*tert*-butyldimethylsilyloxy)-3-phenyl-1-thioxopropan-2-ylcarbamate (**16**). S14

3. Synthesis of heterocyclic core of D-series GE2270 .... S15

a) *tert*-Butyl 2-(2-(2-(1-(*tert*-butyldimethylsilyloxy)vinyl)thiazol-4-yl)-6-(4-(ethoxycarbonyl)thiazol-2-yl)pyridin-3-yl)thiazole-4-carboxylate. S15

b) *tert*-Butyl 2-(2-(2-(2-bromoacetyl)thiazol-4-yl)-6-(4-(ethoxycarbonyl)thiazol-2-yl)pyridin-3-yl)thiazole-4-carboxylate (**17**). S16

c) Synthesis of the central core GE2270 (**18**). S17

III. NMR Spectra S19

1. Synthesis of the trithiazolylpyridine 9 .... S19

a) *tert*-Butyl 2-(2-(2-acetylthiazol-4-yl)-6-carbamoylpyridin-3-yl)thiazole-4-carboxylate (**10**)..... .... S19

2. *tert*-Butyl 2-(2-(2-acetylthiazol-4-yl)-6-carbamothioylpyridin-3-yl)thiazole-4-carboxylate (**11**). S20

3. *tert*-Butyl 2-(2-chloro-6-(4-(ethoxycarbonyl)thiazol-2-yl)pyridine-3-yl)thiazole-4-carboxylate (**7**). S21

4. *tert*-Butyl 2-(2-(2-acetylthiazol-4-yl)-6-(4-(ethoxycarbonyl)thiazol-2-yl)pyridin-3-yl)thiazole-4-carboxylate (**9**). S22

2. Synthesis of chiral thioamide **16** S23

a) (2*S*,3*S*)-Ethyl-2-(benzyloxycarbonylamino)-3-hydroxy-3-phenylpropanoate (**13**). S23

b) (2*S*,3*S*)-Ethyl 2-(benzyloxycarbonylamino)-3-(*tert*-butyldimethylsilyloxy)-3-phenylpropanoate (**14**). S24

c) (2*S*,3*S*)-2-(Benzyloxycarbonylamino)-3-(*tert*-butyldimethylsilyloxy)-3-phenylpropanoic acid (**15**). S25

d) Benzyl (2*S*,3*S*)-1-amino-3-(*tert*-butyldimethylsilyloxy)-3-phenyl-1-thioxopropan-2-ylcarbamate (**16**). S26

3. Synthesis Synthesis of heterocyclic core of D-series GE2270 .... S27

a) *tert*-Butyl 2-(2-(2-(1-(*tert*-butyldimethylsilyloxy)vinyl)thiazol-4-yl)-6-(4-(ethoxycarbonyl)thiazol-2-yl)pyridin-3-yl)thiazole-4-carboxylate S27

b) *tert*-Butyl 2-(2-(2-(2-bromoacetyl)thiazol-4-yl)-6-(4-(ethoxycarbonyl)thiazol-2-yl)pyridin-3-yl)thiazole-4-carboxylate (**17**). S28

4. Synthesis of the central core GE2270A core (**18**). S29

5. HPLC Chromatograms S30

GE2270A core S30

I. General Information

tert-Butyl thiazole-4-carboxylate (**2**),[1] ethyl 5-bromopicolinate (**1**),[2] *tert*-butyl 2-(6-(ethoxycarbonyl)pyridine-3-yl)thiazole-4-carboxylate (**3**),[2] 5-(4-(*tert*-butoxycarbonyl)thiazol-2-yl)-2-(ethoxycarbonyl)pyridine *N*-oxide,[2] *tert*-butyl 2-(2-chloro-6-(ethoxycarbonyl)pyridine-3-yl)thiazole-4carboxylate (**4**),[2] *tert*-butyl 2-(6-carbamoyl-2-chloropyridin-3-yl)thiazole-4-carboxylate (**5**),[2] *tert*-butyl 2-(6-carbamothioyl-2-chloropyridin-3-yl)thiazole-4-carboxylate (**6**),[2] 1-(4-bromothiazol-2-yl)-ethanone (**8**),[3] (2*R*,3*S*)-ethyl 2,3-dihydroxy-3-phenylpropanoate,[4] (2*R*,3*S*)-ethyl 3-hydroxy-2-(4-nitrophenylsulfonyloxy)-3-phenylpropanoate[4] and (2*S*,3*S*)-ethyl 2-azido-3-hydroxy-3-phenylpropanoate (**12**)[4] were prepared according to procedures reported in the literature.

Reactions were performed using oven dried glassware under inert atmosphere of dry argon or nitrogen and monitored by thin-layer chromatography with silica gel 60 F254 pre-coated aluminium plates (0.25 mm). Visualization was performed under UV light and phosphomolybdic acid or $KMnO_4$ oxidation. Chromatographic purification of compounds was achieved with 60 silica gel (40–63 μm).

Solvents and reagents: Toluene and CH_2Cl_2 were dried by refluxing over CaH_2 and then distilled. Unless otherwise noted, all reagent-grade chemicals and solvents were used as supplied (analytical or HPLC grade) without prior purification.

Melting points were measured on a WME Kofler hot-stage with a precision of +/− 2 °C and are uncorrected.

Infrared spectra (IR) were recorded on a PerkinElmer Spectrum 100 Series FT-IR spectrometer. Liquids and solids were applied on the Single Reflection Attenuated Total Reflectance (ATR) Accessories. Data are reported in cm^{-1}.

Optical rotations were determined with a Perkin-Elmer 341 polarimeter with a water-jacketed 10 cm cell. Specific rotations are reported in 10^{-1} deg cm^2 g^{-1} and concentrations in g per 100 mL.

^{1}H Spectra (300 or 400 MHz) and **^{13}C NMR spectra** (75 or 100 MHz) were recorded on either a Bruker Advance300 or Advance400 spectrometers. The field was locked by external referencing to the

[1] Martin, T.; Verrier, C.; Hoarau, C.; Marsais, F. *Org. Lett.* **2008**, *10*, 2909-2912.
[2] Martin, T.; Laguerre, C.; Hoarau, C.; Marsais, F. *Org. Lett.* **2009**, *11*, 3690-3693.
[3] Ung, A. L.; Pyne, S. G. *Tetrahedron: Asymmetry* **1998**, *9*, 1395-1407.
[4] Nicolaou, K. C.; Dethe, D. H.; Leung, G. Y. C.; Zou, B.; Chen, D. Y.-K. *Chem. Asian. J.* **2008**, *3*, 413-429.

relevant deuteron resonance. Data appear in the following order: chemical shifts in ppm which were referenced to the internal solvent signal, number of protons, multiplicity (*s*, singlet; *d*, doublet; *t*, triplet; *dd*, doublet of doublet, *ddd*, doublet of doublet of doublet, *ddt*, doublet of triplet, *m*, multiplet) and coupling constant *J* in Hertz.

Low Resolution Mass Spectra (LRMS) were recorded on a Thermo Electron Corporation ion-trap spectrometer.

Accurate Mass measurements (HRMS) were performed by the Mass Spectrometry Laboratory of the University of Rouen and were recorded with a Waters LCP 1er XR spectrometer.

Elemental analyses were performed by the microanalysis service of the University of Rouen and were recorded with a Thermo Scientific FLASH 2000 analyzer.

HPLC analyses were performed with Daicel Chiralpak® and Chiralcel® columns (4.6 mm × 250 mm) and a mixture of heptane/iPrOH solvents. A spectrosystem UV 1000 thermofisher detector and a chiral detector (polarimeter) JACSCO OR-1590 were used.

II. Experimental Procedures

1. Synthesis of the trithiazolylpyridine 9

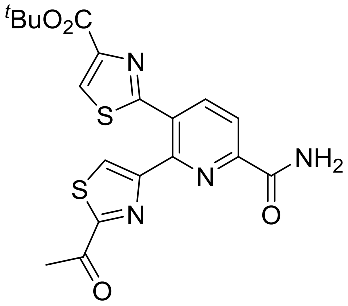

10

a) *tert*-Butyl 2-(2-(2-acetylthiazol-4-yl)-6-carbamoylpyridin-3-yl)thiazole-4-carboxylate (**10**).

Bromothiazole (**8**) (123 mg, 0.60 mmol), bis(pinacolato)diboron (152 mg, 0.60 mmol), $Pd(OAc)_2$ (6 mg, 0.025 mmol), CyJohnPhos (35 mg, 0.10 mmol) and freshly dried KOAc (118 mg, 1.20 mmol) were charged in a Schlenk flask. It was then evacuated and backfilled three times with N_2 and then anhydrous degassed dioxane (2.0 mL) was added and the reaction mixture was stirred at 110 °C in a preheated oil bath for 1 hour. Then the mixture was cooled to room temperature and chloropyridine (**5**) (170 mg, 0.50 mmol), freshly dried K_3PO_4 (531 mg, 2.50 mmol), degassed dioxane (0.5 mL) and degassed water (0.5 mL) were added and the mixture was stirred at 110 °C for further 14 hours. Then the mixture was filtered through a Celite® pad and washed with EtOAc. Water was added and the aqueous layer was extracted three times with EtOAc. The combined organic layers were washed with water brine, dried over anhydrous Na_2SO_4 and concentrated in vacuo. The crude product was purified by flash chromatography on silica gel (eluent: petroleum ether/EtOAc, 1:1 to 3:7) affording **10** (213 mg, 0.49 mmol, 99%) as a colorless solid.

mp = 227-228 °C (decomposition).

IR (ATR diamond): 3440, 3270, 3180, 3080, 2970, 2930, 1710, 1690, 1240, 1160 cm^{-1}.

^{1}H NMR (300 MHz, $CDCl_3$) δ 1.59 (s, 9H, C(CH_3)$_3$), 2.41 (s, 3H, CH_3), 5.69 (br s, 1H, NH_2), 7.80 (br s, 1H, NH_2), 8.15 (s, 1H, $H_{arom.}$), 8.22 (s, 1H, $H_{arom.}$), 8.35 (d, J = 1.7 Hz, 2H, $H_{arom.}$).

^{13}C NMR (75 MHz, $CDCl_3$) δ 25.8 (CH_3), 28.1 (C(CH_3)$_3$), 82.4 (C(CH_3)$_3$), 121.8 ($CH_{arom.}$), 128.0 ($CH_{arom.}$), 128.3 ($CH_{arom.}$), 130.8 ($C_{arom.}$), 140.7 ($CH_{arom.}$), 148.9 ($C_{arom.}$), 149.2 ($C_{arom.}$), 149.8 ($C_{arom.}$), 154.6 ($C_{arom.}$), 160.2 (C=O), 164.5 ($C_{arom.}$), 165.5 (C=O), 166.1 ($C_{arom.}$), 191.4 (C=O).

MS (ESI^+): m/z 430.8 [$(M+H)^+$].

HRMS (ESI^+) Calcd for $C_{19}H_{19}N_4O_4S_2$: 431.0848. Found: 431.0843 [$(M+H)^+$].

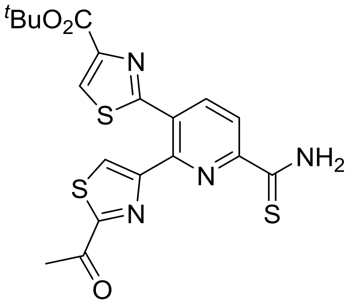

11

b) *tert*-Butyl 2-(2-(2-acetylthiazol-4-yl)-6-carbamothioylpyridin-3-yl)thiazole-4-carboxylate (**11**).

To a stirred solution of amide **10** (200 mg, 0.46 mmol) in anhydrous CH_2Cl_2 (5 mL) was added the Lawesson's reagent (122 mg, 0.30 mmol) and the mixture was heated under reflux for 12 hours. The mixture was then treated by a saturated aqueous $NaHCO_3$ solution. The aqueous layer was extracted three times with EtOAc. The combined organic layers were washed with brine, dried over anhydrous Na_2SO_4 and concentrated in vacuo. The crude product was quickly purified by flash chromatography on silica gel (eluent: petroleum ether/EtOAc, 9:1 to 1:1) affording **11** (187 mg, 0.42 mmol, 91%) as a colorless solid.

mp 188-189 °C.

IR (ATR diamond): 3330, 3270, 3140, 3090, 2980, 2940, 1720, 1690, 1160 cm^{-1}.

^{1}H NMR (300 MHz, $CDCl_3$) δ 1.59 (s, 9H, $C(CH_3)_3$), 2.43 (s, 3H, CH_3), 7.77 (br s, 1H, NH_2), 8.15 (s, 1H, H_{arom}), 8.18 (s, 1H, H_{arom}), 8.34 (d, J = 8.2 Hz, 1H, H_{arom}), 8.81 (d, J = 8.3 Hz, 1H, $H_{arom.}$), 9.40 (br s, 1H, NH_2).

^{13}C NMR (75 MHz, $CDCl_3$) δ 25.9 (CH_3), 28.3 ($C(CH_3)_3$), 82.6 ($C(CH_3)_3$), 124.5 ($CH_{arom.}$), 128.3 ($CH_{arom.}$), 128.5 ($CH_{arom.}$), 130.5 ($C_{arom.}$), 140.3 ($CH_{arom.}$), 148.2 ($C_{arom.}$), 148.9 ($C_{arom.}$), 150.5 ($C_{arom.}$), 154.6 ($C_{arom.}$), 160.4 (C=O), 164.7 ($C_{arom.}$), 166.2 ($C_{arom.}$), 191.5 (C=O), 194.2 (C=S).

MS (ESI^+): m/z 446.9 [$(M+H)^+$].

HRMS (ESI^+) Calcd for $C_{19}H_{18}N_4O_3S_3Na$: 469.0433. Found: 469.0428 [$(M+Na)^+$].

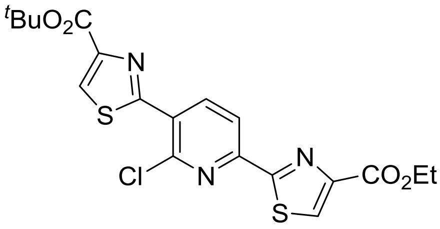

7

c) *tert*-Butyl 2-(2-chloro-6-(4-(ethoxycarbonyl)thiazol-2-yl)pyridine-3-yl)thiazole-4-carboxylate (**7**).

To a stirred solution of thioamide **6** (480 mg, 1.35 mmol) in a mixture of anhydrous THF/EtOH (1:1, 10 mL) were added $CaCO_3$ (405 g, 4.00 mmol) and ethyl bromopyruvate (250 µL, 2.00 mmol) and the mixture was stirred at 60 °C for 24 hours. Then water was added and the aqueous layer was extracted three times with EtOAc. The combined organic layers were washed with brine, dried over anhydrous Na_2SO_4 and concentrated in vacuo. The crude product was purified by flash chromatography on silica gel (eluent: petroleum ether/EtOAc, 95:3 to 7:3) affording **7** (350 g, 0.78 mmol, 72%) as a colorless solid.

mp 188-189 °C.

IR (ATR diamond): 3120, 2980, 2940, 1730, 1700 cm^{-1}.

^{1}H NMR (300 MHz, $CDCl_3$) δ 1.45 (t, J = 7.1 Hz, 3H, CH_3), 1.64 (s, 9H, $C(CH_3)_3$), 4.47 (q, J = 7.1 Hz, 2H, CH_2), 8.24 (s, 1H, $H_{arom.}$), 8.33 (s, 1H, $H_{arom.}$), 8.39 (d, J = 8.2 Hz, 1H, $H_{arom.}$), 8.97 (d, J = 8.2 Hz, 1H, $H_{arom.}$).

^{13}C NMR (75 MHz, $CDCl_3$) δ 14.3 (CH_3), 28.1 ($C(CH_3)_3$), 61.7 (CH_2), 82.5 ($C(CH_3)_3$), 119.1 ($CH_{arom.}$), 128.5 ($CH_{arom.}$), 129.1 ($C_{arom.}$), 130.5 ($CH_{arom.}$), 140.8 ($CH_{arom.}$), 147.3 ($C_{arom.}$), 148.7 ($C_{arom.}$), 148.7 ($C_{arom.}$), 150.8 ($C_{arom.}$), 160.2 (C=O), 161.1 ($C_{arom.}$), 161.2 (C=O), 167.0 ($C_{arom.}$).

MS (ESI^+): m/z 451.9 and 453.8 [$(M+H)^+$].

Anal. Calcd for $C_{19}H_{18}ClN_3O_4S_2$: C, 50.49; H, 4.01; N, 9.30. Found: C, 50.21; H, 4.08; N, 9.34.

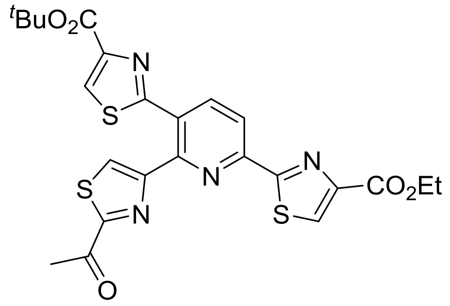

9

d) *tert*-Butyl 2-(2-(2-acetylthiazol-4-yl)-6-(4-(ethoxycarbonyl)thiazol-2-yl)pyridin-3-yl)thiazole-4-carboxylate (9).

Hantzsch thiazole synthesis: To a stirred solution of thioamide **11** (113 mg, 0.25 mmol) in a mixture of anhydrous THF/EtOH (1:1, 3 mL) were added $CaCO_3$ (50 mg, 0.75 mmol) and ethyl bromopyruvate (50 μL, 0.28 mmol) and the mixture was stirred at 60 °C for 24 hours. It was then *concentrated in vacuo*, water was added and the aqueous layer was extracted three times with EtOAc. The combined organic layers were washed brine, dried over anhydrous Na_2SO_4 and concentrated in vacuo. The crude product was purified by flash chromatography on silica gel (eluent: petroleum ether/EtOAc, 95:3 to 7:3) affording **9** (88 mg, 0.16 mmol, 65%) as a colorless solid.

Palladium-catalyzed BSC (borylation–Suzuki coupling) procedure: 4-Bromo-2acetylthiazole (**8**) (26 mg, 0.13 mmol), bis(pinacolato)diboron (32 mg, 0.13 mmol), $Pd(OAc)_2$ (1.2 mg, 0.005 mmol), CyJohnPhos (7.3 mg, 0.021 mmol) and freshly dried KOAc (25 mg, 0.26 mmol) were charged in a Schlenk flask. It was then evacuated and backfilled three times with N_2 and then anhydrous degassed dioxane (0.45 mL) was added and the reaction mixture was stirred at 110 °C in a preheated oil bath for 1 hour. Then the mixture was cooled to room temperature and chloropyridine (**7**) (47 mg, 0.10 mmol), freshly dried K_3PO_4 (111 mg, 0.52 mmol), degassed dioxane (0.11 mL) and degassed water (0.11 mL) were added and the mixture was stirred at 110 °C for further 14 hours. Then the mixture was filtered through a Celite® pad and washed with EtOAc. Water was added and the aqueous layer was extracted three times with EtOAc. The combined organic layers were washed with water brine, dried over anhydrous Na_2SO_4 and concentrated in vacuo. The crude product was purified by flash chromatography on silica gel (eluent: petroleum ether/EtOAc, 6:4) affording **9** (47 mg, 0.087 mmol, 82%) as a colorless solid.

mp 180-181 °C.

IR (ATR diamond): 3100, 2980, 2940, 1710, 1680, 1230 cm^{-1}.

^{1}H NMR (300 MHz, $CDCl_3$) δ 1.46 (t, J = 7.0 Hz, 3H, CH_3), 1.60 (s, 9H, $C(CH_3)_3$), 2.38 (s, 3H, CH_3), 4.48 (q, J = 7.1 Hz, 2H, CH_2), 8.16 (s, 1H, $H_{arom.}$), 8.27 (d, J = 8.1 Hz, 1H, $H_{arom.}$), 8.33 (s, 1H, $H_{arom.}$), 8.40 (s, 1H, $H_{arom.}$), 8.43 (d, J = 8.2 Hz, 1H, $H_{arom.}$).

^{13}C NMR (75 MHz, $CDCl_3$) δ 14.5 (CH_3), 25.9 (CH_3), 28.3 ($C(CH_3)_3$), 61.8 (CH_2), 82.4 ($C(CH_3)_3$), 119.4 ($CH_{arom.}$), 128.2 ($CH_{arom.}$), 128.4 ($CH_{arom.}$), 129.2 ($C_{arom.}$), 130.3 ($CH_{arom.}$), 140.7 ($CH_{arom.}$), 148.8 ($C_{arom.}$), 148.9 ($C_{arom.}$), 150.0 ($C_{arom.}$), 150.9 ($C_{arom.}$), 154.9 ($C_{arom.}$), 160.4 (C=O), 161.4 (C=O), 165.1 ($C_{arom.}$) 166.0 ($C_{arom.}$) 168.6 ($C_{arom.}$) 191.6 (C=O).

MS (ESI^+): *m/z* 543.0 [$(M+H)^+$].

Anal. Calcd for $C_{24}H_{22}N_4O_5S_3$: C, 53.12; H, 4.09; N, 10.32. Found: C, 52.89; H, 4.43; N, 10.41.

2. Synthesis of chiral thioamide 16

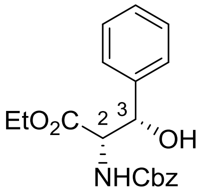

13

a) (2*S*,3*S*)-Ethyl 2-(benzyloxycarbonylamino)-3-hydroxy-3-phenylpropanoate (**13**).

To a stirred solution of azide **12** (2.4 g, 10.2 mmol) in a mixture of dioxane/H_2O (1:3, 80 mL) at 0 °C was slowly added tin(II) chloride dihydrate (11.5 g, 50.1 mmol). Then the mixture was allowed to warm to room temperature and stirred for 5 hours. The mixture was then carefully treated at 0 °C with a saturated aqueous $NaHCO_3$ solution to reach pH 8–9 after which benzyl chloroformate (2.2 mL, 15.0 mmol) was added. The mixture was stirred at room temperature for further 18 hours. Then the mixture was carefully hydrolyzed with HCl (3N), filtered through a Celite® pad. The filtrate was extracted three times with EtOAc. The combined organic layers were washed with brine, dried over anhydrous Na_2SO_4 and concentrated in vacuo. The crude product was purified by flash chromatography on silica gel (eluent: petroleum ether/EtOAc, 9:1 to 7:3) affording **13** (3.9 g, 11.6 mmol, 96%) as a white solid.

mp 74-75 °C.

$[\alpha]_D^{20}$ +63.3 (*c* 3.04, $CHCl_3$).

IR (ATR diamond): 3330, 3070, 3040, 2980, 2960, 2900, 1740, 1690, 1540 cm^{-1}.

^{1}H NMR (300 MHz, $CDCl_3$) δ 1.17 (t, *J* = 7.1 Hz, 3H, CH_3), 3.54 (d, *J* = 4.4 Hz, 1H, OH), 4.13 (q, *J* = 7.1 Hz, 2H, CH_2), 4.76 (dd, *J* = 7.8 Hz, 3.9 Hz, 1H, H_2), 5.12-5.13 (m, 2H, CH_2Ph), 5.22 (m, 1H, H_3), 5.53 (d, *J* = 7.1 Hz, 1H, NH), 7.24-7.41 (m, 10H, $H_{arom.}$).

^{13}C NMR (75 MHz, $CDCl_3$) δ 14.0 (CH_3), 60.1 (C_2), 61.8 (CH_2), 67.4 (CH_2Ph), 74.8 (C_3), 126.1 ($CH_{arom.}$), 128.2 ($CH_{arom.}$), 128.2 ($CH_{arom.}$), 128.4 ($CH_{arom.}$), 128.4 ($CH_{arom.}$), 128.6 ($CH_{arom.}$), 136.1 ($C_{arom.}$), 139.1 ($C_{arom.}$), 156.8 (C=O), 169.7 (C=O).

MS (ESI^+): *m/z* 344.0 [$(M+H)^+$].

Anal. Calcd for $C_{19}H_{21}NO_5$: C, 66.46; H, 6.16; N, 4.08. Found: C, 66.47; H, 6.31; N, 4.21.

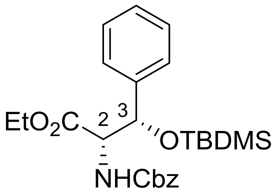

14

b) (2*S*,3*S*)-Ethyl 2-(benzyloxycarbonylamino)-3-(*tert*-butyldimethylsilyloxy)-3-phenylpropanoate (**14**).

To a stirred solution of amino alcohol **13** (1.90 g, 5.53 mmol) in anhydrous DMF (11 mL) were added imidazole (4.13 g, 16.59 mmol) and TBDMSCl (1.67 g, 11.08 mmol) and the mixture was stirred for 16 hours. Then the mixture was concentrated in vacuo. Water was added and the aqueous layer was extracted three times with EtOAc. The combined organic layers were washed with water, twice with brine, dried over anhydrous Na_2SO_4 and concentrated in vacuo. The crude product was purified by flash chromatography on silica gel (eluent: petroleum ether/EtOAc, 95:5 to 85:15) affording **14** (2.50 g, 5.46 mmol, 99%) as a colorless oil.

$[\alpha]_D^{20}$ +54.1 (*c* 1.05, $CHCl_3$).

IR (ATR diamond): 3450, 3360, 3070, 3030, 2960, 2930, 2900, 2850, 1720, 1500 cm^{-1}.

^{1}H NMR (300 MHz, $CDCl_3$) δ –0.12 (s, 3H, CH_3), –0.06 (s, 3H, CH_3), 0.91 (s, 9H, $C(CH_3)_3$), 1.14 (t, *J* = 7.1 Hz, 3H, CH_3), 4.08 (q, *J* = 7.1 Hz, 2H, CH_2), 4.61 (dd, *J* = 8.4 Hz, 4.0 Hz, 1H, H_2), 5.11 (m, 3H, H_3, CH_2Ph), 5.51 (d, *J* = 8.3 Hz, 1H, NH), 7.27-7.40 (m, 10H, $H_{arom.}$).

^{13}C NMR (75 MHz, $CDCl_3$) δ –5.4 (CH_3), –4.9 (CH_3), 13.9 (CH_3), 18.0 ($C(CH_3)_3$), 25.6 $C(CH_3)_3$), 61.1 (CH_2), 61.2 (C_2), 66.8 (CH_2Ph), 75.3 (C_3), 126.2 ($CH_{arom.}$), 127.7 ($CH_{arom.}$), 128.0 ($CH_{arom.}$), 128.0 ($CH_{arom.}$), 128.1 ($CH_{arom.}$), 128.4 ($CH_{arom.}$), 136.3 ($C_{arom.}$), 140.2 ($C_{arom.}$), 155.5 (C=O), 169.4 (C=O).

MS (ESI^+): *m/z* 458.1 [$(M+H)^+$], 936.9 [$(M_2+Na)^+$].

Anal. Calcd for $C_{25}H_{35}NO_5Si$: C, 65.61; H, 7.71; N, 3.06. Found: C, 65.65; H, 7.67; N, 3.51.

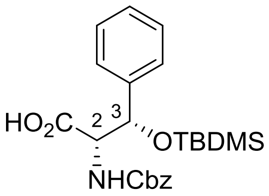

15

c) (2*S*,3*S*)-2-(Benzyloxycarbonylamino)-3-(*tert*-butyldimethylsilyloxy)-3-phenylpropanoic acid (**15**).

To a stirred solution of ester **14** (2.50 g, 5.46 mmol) in a mixture of DME/H_2O (1:1, 30 mL) at 0 °C was added lithium hydroxide monohydrate (0.32 g, 7.64 mmol). Then the mixture was allowed to warm to room temperature and stirred for 72 hours. The mixture was then carefully treated at 0 °C with HCl (3 N). The aqueous layer was extracted three times with EtOAc. The combined organic layers were washed with brine, dried over anhydrous Na_2SO_4 and concentrated in vacuo. The crude product was crushed with pentane and filtered to afford **15** (2.22 g, 5.17 mmol, 96%) as a white solid.

mp 55-56 °C.

$[\alpha]_D^{20}$ +61.2 (*c* 0.97, $CHCl_3$).

IR (ATR diamond): 3430, 3300, 3060, 3030, 2960, 2930, 2900, 2860, 1710, 1690 cm^{-1}.

^{1}H NMR (300 MHz, $CDCl_3$) δ –0.12 (s, 3H, CH_3), –0.05 (s, 3H, CH_3), 0.89 (s, 9H, C(CH_3)$_3$), 4.66 (dd, J = 8.4 Hz, 4.2 Hz, 1H, H_2), 5.10-5.13 (m, 3H, H_3, CH_2Ph), 5.13 (d, J = 4.2 Hz, 1H, NH), 7.27-7.38 (m, 10H, $H_{arom.}$).

^{13}C NMR (75 MHz, $CDCl_3$) δ –5.2 (CH_3), –4.8 (CH_3), 18.2 (*C*(CH_3)$_3$), 25.8 C(CH_3)$_3$), 61.3 (C_2), 67.2 (CH_2Ph), 75.2 (C_3), 126.4 ($CH_{arom.}$), 128.2 ($CH_{arom.}$), 128.3 ($CH_{arom.}$), 128.3 ($CH_{arom.}$), 128.6 ($CH_{arom.}$), 136.3 ($C_{arom.}$), 139.7 ($C_{arom.}$), 155.8 (C=O), 174.8 (C=O).

MS (ESI^+): *m/z* 429.9 [$(M+H)^+$], 880.8 [$(M_2+Na)^+$].

Anal. Calcd for $C_{23}H_{31}NO_5Si$: C, 64.31; H, 7.27; N, 3.26. Found: C, 64.34; H, 7.38; N, 3.30.

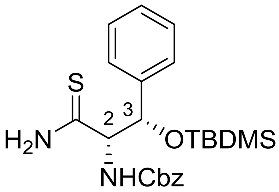

16

d) Benzyl (2*S*,3*S*)-1-amino-3-(*tert*-butyldimethylsilyloxy)-3-phenyl-1-thioxopropan-2-ylcarbamate (**16**).

To a stirred solution of acid **15** (2.15 g, 5.00 mmol) in anhydrous THF (25 mL) at 0 °C were added DCC (1.28 g, 6.21 mmol) and *N*-hydroxysuccinimide (0.71 g, 6.21 mmol). The mixture was allowed to warm to room temperature and stirred for 16 hours. Then the mixture was filtered through a Celite® pad and concentrated in vacuo. The resulting residue was dissolved in AcOEt (25 mL) and cooled to 0 °C. An ammonium hydroxide solution (30% in H_2O, 7 mL) was slowly added then the mixture was allowed to warm to room temperature and stirred for 3 hours. Then the aqueous layer was extracted three times with EtOAc. The combined organic layers were washed with water, brine, dried over anhydrous Na_2SO_4 and concentrated in vacuo. To a stirred solution of crude amide in anhydrous DME (13 mL) was added the Lawesson's reagent (1.3 g, 3.26 mmol) and the mixture was stirred at room temperature for 36 hours before it was filtered through a Celite® pad and concentrated in vacuo. The crude product was purified by flash chromatography on silica gel (eluent: petroleum ether/Et_2O, 9:1 to 1:1) affording **16** (1.60 g, 3.60 mmol, 72% over three steps) as a white solid.

mp 75-76 °C.

$[\alpha]_D^{20}$ +22.3 (*c* 0.98, $CHCl_3$).

HPLC: Chiralpak IA (heptane/isopronol, 7/3), λ = 254 nm, 1 mL.min^{-1}. t_R = 4.80 min. *ee* > 99%.

IR (ATR diamond): 3300, 3200, 3070, 3040, 2950, 2930, 2890, 2850, 1700, 1630, 1500 cm^{-1}.

^{1}H NMR (300 MHz, CD_3CN) δ –0.24 (s, 3H, CH_3), –0.03 (s, 3H, CH_3), 0.83 (s, 9H, $C(CH_3)_3$), 4.56 (dd, *J* = 9.7 Hz, 8.2 Hz, 1H, H_2), 4.82-4.95 (m, 3H, H_3, CH_2Ph), 5.77-5.80 (m, 1H, NH), 7.14-7.18 (m, 2H, $H_{arom.}$), 7.27-7.36 (m, 6H, $H_{arom.}$), 7.41-7.45 (m, 2H, $H_{arom.}$), 8.09-8.21 (m, 2H, NH_2).

^{13}C NMR (75 MHz, CD_3CN) δ –4.3 (CH_3), –4.0 (CH_3), 19.3 ($C(CH_3)_3$), 26.6 $C(CH_3)_3$), 67.6 (C_2), 67.6 (CH_2Ph), 78.6 (C_3), 129.0 ($CH_{arom.}$), 129.1 ($CH_{arom.}$), 129.4 ($CH_{arom.}$), 129.6 ($CH_{arom.}$), 130.0 ($CH_{arom.}$), 138.6 ($C_{arom.}$), 142.7 ($C_{arom.}$), 156.7 (C=O), 208.4 (C=S).

MS (ESI$^+$): *m/z* 444.9 [$(M+H)^+$], 888.7 [$(M_2+H)^+$].

Anal. Calcd for $C_{23}H_{32}N_2O_3SSi$: C, 62.12; H, 7.25; N, 6.30. Found: C, 61.83; H, 7.40; N, 6.66.

3. Synthesis of heterocyclic core of D-series GE2270

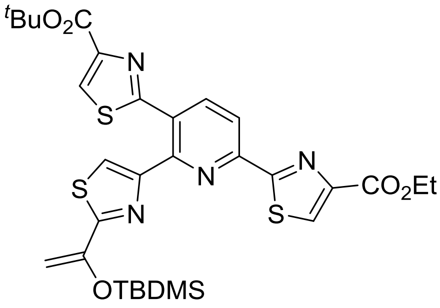

a) *tert*-Butyl 2-(2-(2-(1-(*tert*-butyldimethylsilyloxy)vinyl)thiazol-4-yl)-6-(4-(ethoxycarbonyl)thiazol-2-yl)pyridin-3-yl)thiazole-4-carboxylate.

To a stirred solution of ketone **9** (40 mg, 0.074 mmol) in anhydrous CH_2Cl_2 (0.4 mL) at 0 C were added NEt_3 (34 µL, 0.24 mmol) and *tert*-butyldimethylsilyl trifluoromethanesulfonate (38 µL, 0.16 mmol) and the mixture was stirred for 1 hour. Then water was added and the aqueous layer was extracted three times with CH_2Cl_2. The combined organic layers were washed with brine, dried over anhydrous $MgSO_4$ and concentrated in vacuo. The crude product was purified by flash chromatography on silica gel (pre-treated with 3% of triethylamine, eluent: petroleum ether/EtOAc, 7:3) affording the title compound (24 mg, 0.037 mmol, 51%) as a white solid.

mp 188-189 °C.

IR (ATR diamond): 3100, 2980, 2940, 1730, 1210 cm^{-1}.

^{1}H NMR (300 MHz, $CDCl_3$) δ 0.24 (s, 6H, CH_3), 1.01 (s, 9H, C(CH_3)$_3$), 1.45 (t, J = 7.1 Hz, 3H, CH_3), 1.60 (s, 9H, C(CH_3)$_3$), 4.37 (d, J = 1.0 Hz, 1H, H_a of $CH_{2\ vinyl}$), 4.47 (q, J = 7.1 Hz, 2H, CH_2), 5.12 (d, J = 1.5 Hz, 1H, H_b of $CH_{2\ vinyl}$), 7.90 (s, 1H, $H_{arom.}$), 8.10 (s, 1H, $H_{arom.}$), 8.30 (s, 1H, $H_{arom.}$), 8.39 (s, 2H, $H_{arom.}$).

^{13}C NMR (75 MHz, $CDCl_3$) δ –4.6 (CH_3), 14.5 (CH_3), 18.3 (C(CH_3)$_3$), 25.8 (C(CH_3)$_3$), 28.3 (C(CH_3)$_3$), 61.8 (CH_2), 82.2(C(CH_3)$_3$), 92.3 ($CH_{2\ vinyl}$), 119.2 ($CH_{arom.}$), 121.8 ($CH_{arom.}$), 128.5 ($CH_{arom.}$), 129.5 ($C_{arom.}$), 130.3 ($CH_{arom.}$), 140.3 ($CH_{arom.}$), 148.5 ($C_{arom.}$), 148.6 ($C_{arom.}$), 149.4 (C_{vinyl}), 150.6 ($C_{arom.}$), 151.0 ($C_{arom.}$), 153.6 ($C_{arom.}$), 160.6 (C=O), 161.5 (C=O), 165.1 ($C_{arom.}$), 167.1 ($C_{arom.}$), 169.1 ($C_{arom.}$).

MS (ESI^+): m/z 657.0 [$(M+H)^+$], 1312.5 [$(M_2+H)^+$].

HRMS (ESI^+) Calcd for $C_{30}H_{36}N_4O_5S_3SiNa$: 679.1515. Found: 679.1514 [$(M+Na)^+$].

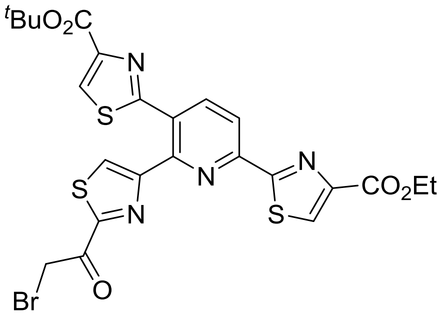

17

b) *tert*-Butyl 2-(2-(2-(2-bromoacetyl)thiazol-4-yl)-6-(4-(ethoxycarbonyl)thiazol-2-yl)pyridin-3-yl)thiazole-4-carboxylate (**17**).

To a stirred solution of enol previously prepared (40 mg, 610 μmol) in anhydrous THF (2 mL) at 0 °C was added *N*-bromosuccinimide (11 mg, 61 μmol) and the mixture was stirred for 1 hour. Then water was added and the aqueous layer was extracted three times with Et_2O. The combined organic layers were washed with brine, dried over anhydrous Na_2SO_4 and concentrated in vacuo. The crude product was purified by flash chromatography on silica gel (eluent: petroleum ether/EtOAc, 95:3 to 1:1) affording **17** (36 mg, 58 μmol, 94%) as a colorless solid.

mp 190-191 °C.

IR (ATR diamond): 3100, 2980, 2940, 1710, 1240 cm^{-1}.

^{1}H NMR (300 MHz, $CDCl_3$) δ 1.44 (t, J = 7.1 Hz, 3H, CH_3), 1.59 (s, 9H, $C(CH_3)_3$), 4.25 (s, 2H, CH_2), 4.46 (q, J = 7.1 Hz, 2H, CH_2), 8.19-8.21 (m, 2H, $H_{arom.}$), 8.32 (s, 1H, $H_{arom.}$), 8.42 (d, J = 8.1 Hz, 1H, $H_{arom.}$), 8.53 (s, 1H, $H_{arom.}$).

^{13}C NMR (75 MHz, $CDCl_3$) δ 14.5 (CH_3), 28.3 ($C(CH_3)_3$), 30.4 (CH_2), 61.8 (CH_2), 82.6 ($C(CH_3)_3$), 119.5 ($CH_{arom.}$), 128.3 ($CH_{arom.}$), 129.2 ($C_{arom.}$), 129.6 ($CH_{arom.}$), 130.4 ($CH_{arom.}$), 140.8 ($CH_{arom.}$), 148.8 ($C_{arom.}$), 149.0 ($C_{arom.}$), 149.5 ($C_{arom.}$), 151.0 ($C_{arom.}$), 155.3 ($C_{arom.}$), 160.3 (C=O), 161.4 (C=O), 162.6 ($C_{arom.}$), 165.1 ($C_{arom.}$), 168.5 ($C_{arom.}$), 184.6 (C=O).

MS (ESI^+): m/z 620.9 and 622.8 [$(M+H)^+$].

HRMS (ESI^+) Calcd for $C_{24}H_{21}BrN_4O_5S_3Na$: 642.9755 and 644.9735. Found: 642.9758 and 644.9750 [$(M+Na)^+$].

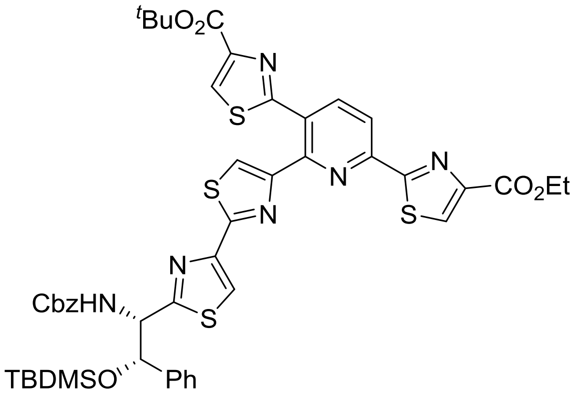

18

c) Synthesis of the central core GE2270 (**18**).

To a stirred solution of thioamide **16** (22 mg, 49 μmol) in anhydrous DMF (2 mL) at –20 °C were added MS 4° (40 mg) and bromoketone **17** (25 mg, 40 μmol). The mixture was allowed to warm to 0 °C and was stirred at this temperature for 14 hours. Then the mixture was filtered through a Celite® pad and concentrated in vacuo. The crude product was purified by flash chromatography on silica gel (eluent: petroleum ether/EtOAc, 9:1 to 1:1) affording the title compound (31 mg, 31 μmol, 78%) which was dissolved in anhydrous DME (1 mL) and cooled at –40 °C before 2,6-lutidine (50 μL, 0.40 mmol) and trifluoroacetic anhydride (20 μL, 0.15 mmol) were added. The mixture was allowed to warm to –20 °C and stirred at this temperature for 12 hours. Then NEt_3 was added until pH 8-9, water was added and the aqueous layer was extracted three times with CH_2Cl_2. The combined organic layers were washed with brine, dried over anhydrous Na_2SO_4 and concentrated in vacuo. The crude product was purified by flash chromatography on silica gel (eluent: petroleum ether/EtOAc, 95:5 to 8:2) affording **18** (26 mg, 27 μmol, 85% over two steps) as a yellow solid.

mp 119-120 °C.

$[\alpha]_D^{20}$ –10.2 (*c* 1.05, $CHCl_3$).

HPLC: Chiralpak IA (heptane/isopronol, 8/2), λ = 254 nm, 1 mL.min^{-1}. t_R (major) = 27.86 min, t_R (minor) = 32.81 min. *ee* > 99%, dr = 92:8.

IR (ATR diamond): 3430, 3330, 3120, 3050, 3020, 2960, 2930, 2900, 2860, 1710, 1500, 1370, 1330, 1250, 1200, 1160, 1100, 1020 cm^{-1}.

^{1}H NMR (300 MHz, $CDCl_3$) δ –0.17 (s, 3H, CH_3), –0.02 (s, 3H, CH_3), 0.85 (s, 9H, $C(CH_3)_3$), 1.46 (t, J = 7.1 Hz, 3H, CH_3), 1.59 (s, 9H, $C(CH_3)_3$), 4.48 (q, J = 7.01 Hz, 2H, CH_2), 5.04-5.13 (m, 2H, CH_2Ph), 5.24-5.31 (m, 2H, H_1, H_2), 5.73 (d, J = 7.9 Hz, 1H, NH), 7.33 (br s, 10H, $H_{arom.}$), 7.49 (s, 1H, $H_{arom.}$), 7.95 (s, 1H, $H_{arom.}$), 8.09 (s, 1H, $H_{arom.}$), 8.32 (s, 1H, $H_{arom.}$), 8.42 (s, 2H, $H_{arom.}$).

^{13}C NMR (75 MHz, $CDCl_3$) δ –5.2 ($SiCH_3$), –4.7 ($SiCH_3$), 14.5 (CH_3), 18.2 (Cq, $C(CH_3)_3$), 25.9 ($C(CH_3)_3$), 28.3 ($C(CH_3)_3$), 59.8 (C_1), 61.8 (CH_2), 67.2 (CH_2Ph), 77.4 (C_2), 82.4 (Cq, $C(CH_3)_3$), 116.4 ($CH_{arom.}$), 119.3

($CH_{arom.}$), 122.0 ($CH_{arom.}$), 126.8 ($CH_{arom.}$), 128.1 ($CH_{arom.}$), 128.3 ($CH_{arom.}$), 128.3 ($CH_{arom.}$), 128.4 ($CH_{arom.}$), 128.5 ($CH_{arom.}$), 128.7 ($CH_{arom.}$), 129.5 ($C_{arom.}$), 130.3 ($CH_{arom.}$), 136.3 ($C_{arom.}$), 139.9 ($C_{arom.}$), 140.3 ($CH_{arom.}$), 148.5 ($C_{arom.}$), 148.6 ($C_{arom.}$), 148.7 ($C_{arom.}$), 150.7 ($C_{arom.}$), 150.9 ($C_{arom.}$), 153.6 ($C_{arom.}$), 155.5 (C=O), 160.6 (C=O), 161.5 (C=O), 162.3 ($C_{arom.}$), 165.3 ($C_{arom.}$), 167.8 ($C_{arom.}$), 169.1 ($C_{arom.}$).

MS (ESI^+): *m/z* 966.8 [$(M+H)^+$], 988.9 [$(M+Na)^+$], 1949.9 [$(M+NH_4)^+$].

HRMS (ESI^+) Calcd for $C_{47}H_{51}N_6O_7S_4Si$: 967.2466. Found: 967.2461 [$(M+H)^+$].

III. NMR Spectra

1. Synthesis of the trithiazolylpyridine 9

a) *tert*-Butyl 2-(2-(2-acetylthiazol-4-yl)-6-carbamoylpyridin-3-yl)thiazole-4-carboxylate (**10**).

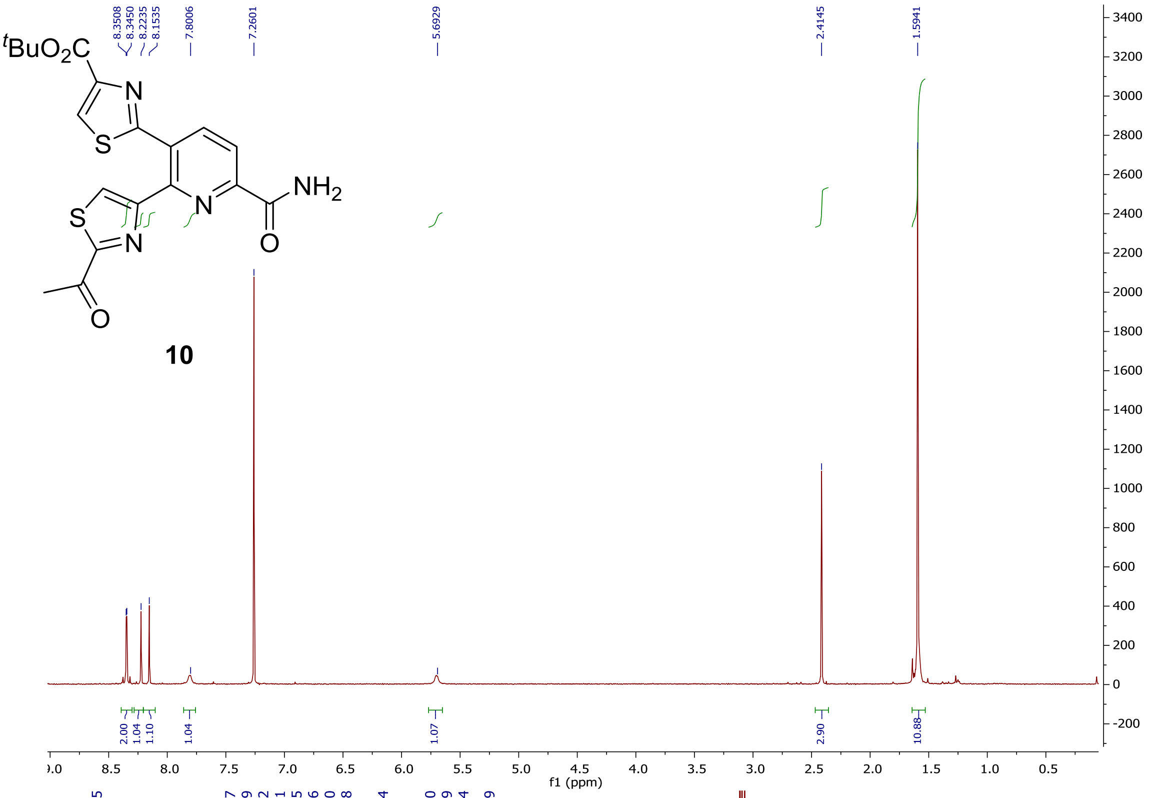

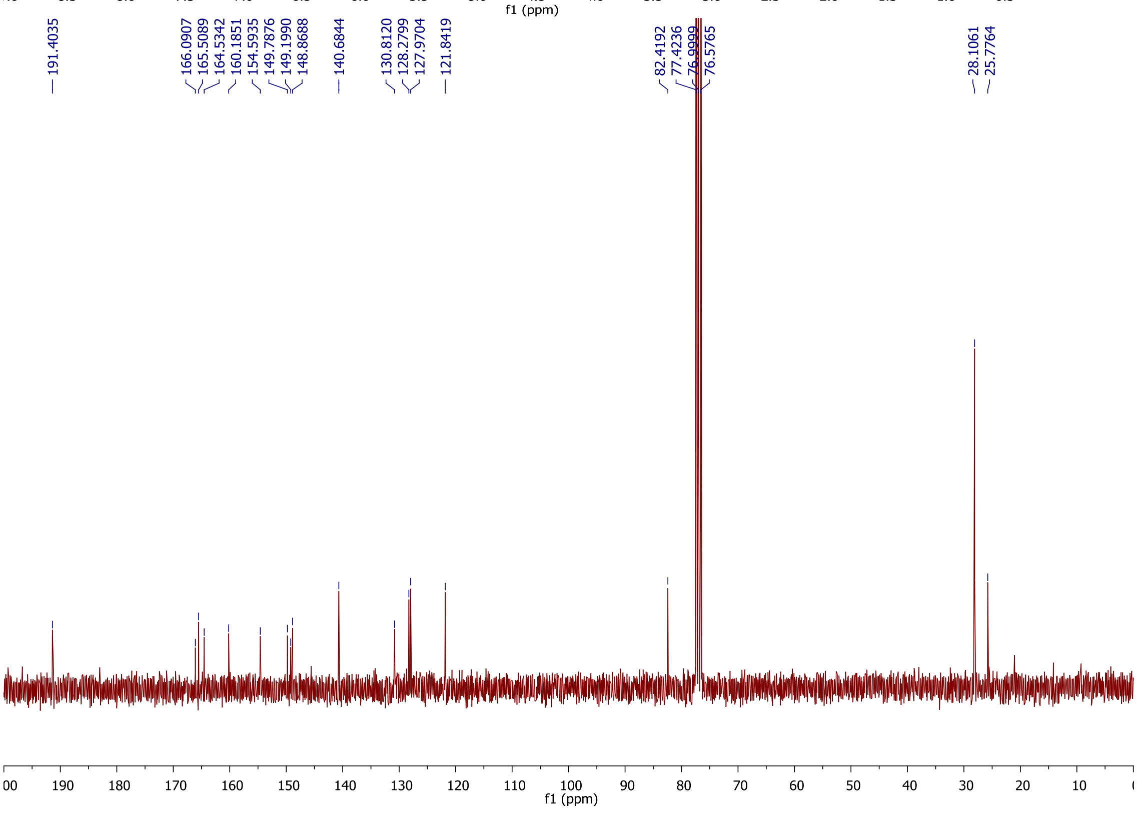

2. *tert*-Butyl 2-(2-(2-acetylthiazol-4-yl)-6-carbamothioylpyridin-3-yl)thiazole-4-carboxylate (**11**).

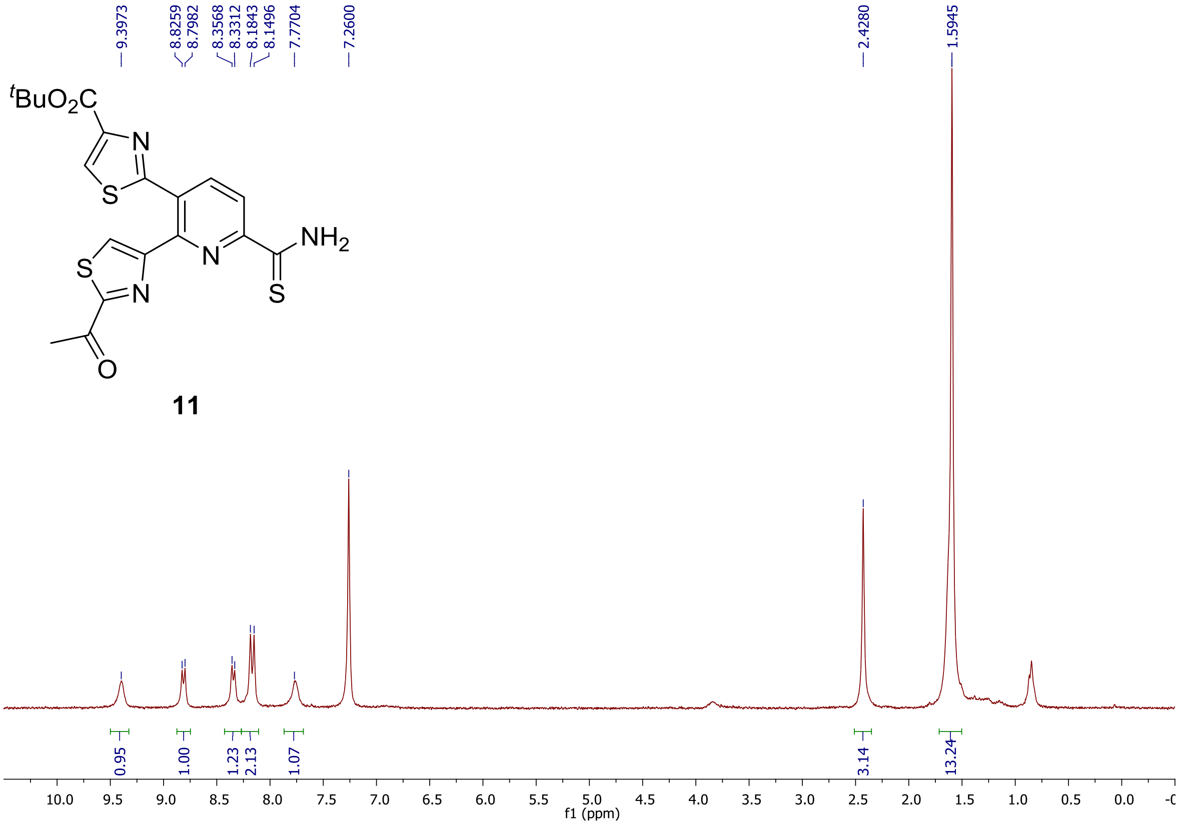

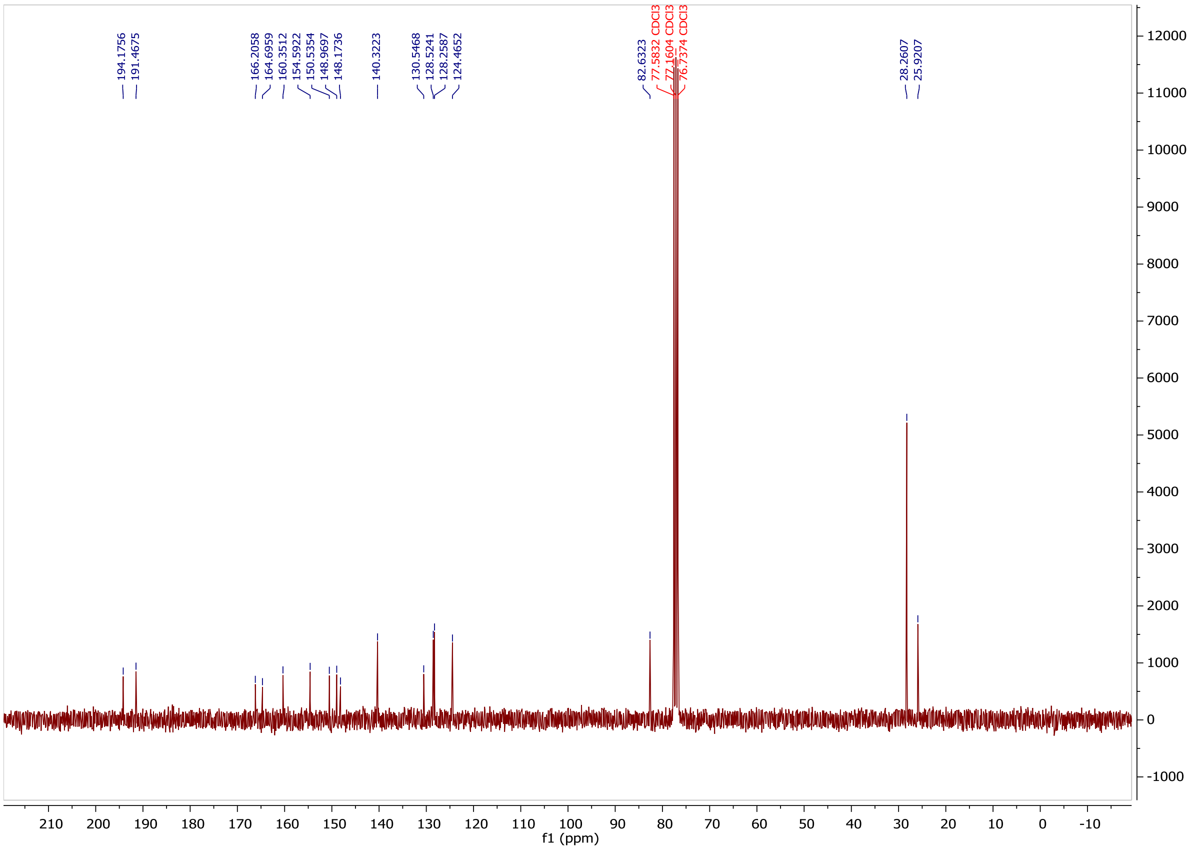

3. *tert*-Butyl 2-(2-chloro-6-(4-(ethoxycarbonyl)thiazol-2-yl)pyridine-3-yl)thiazole-4-carboxylate (**7**).

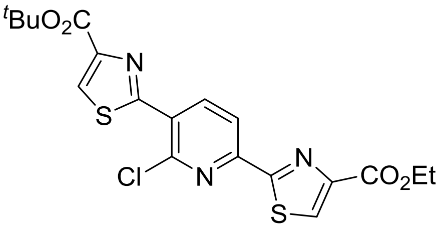

7

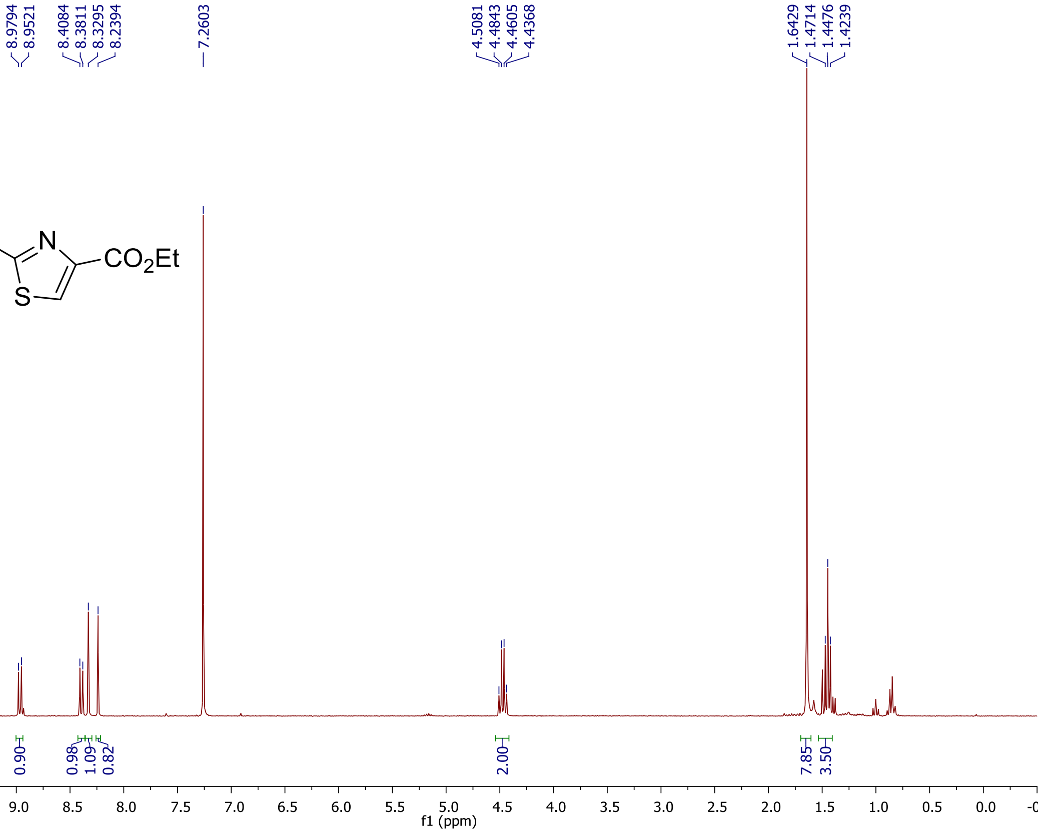

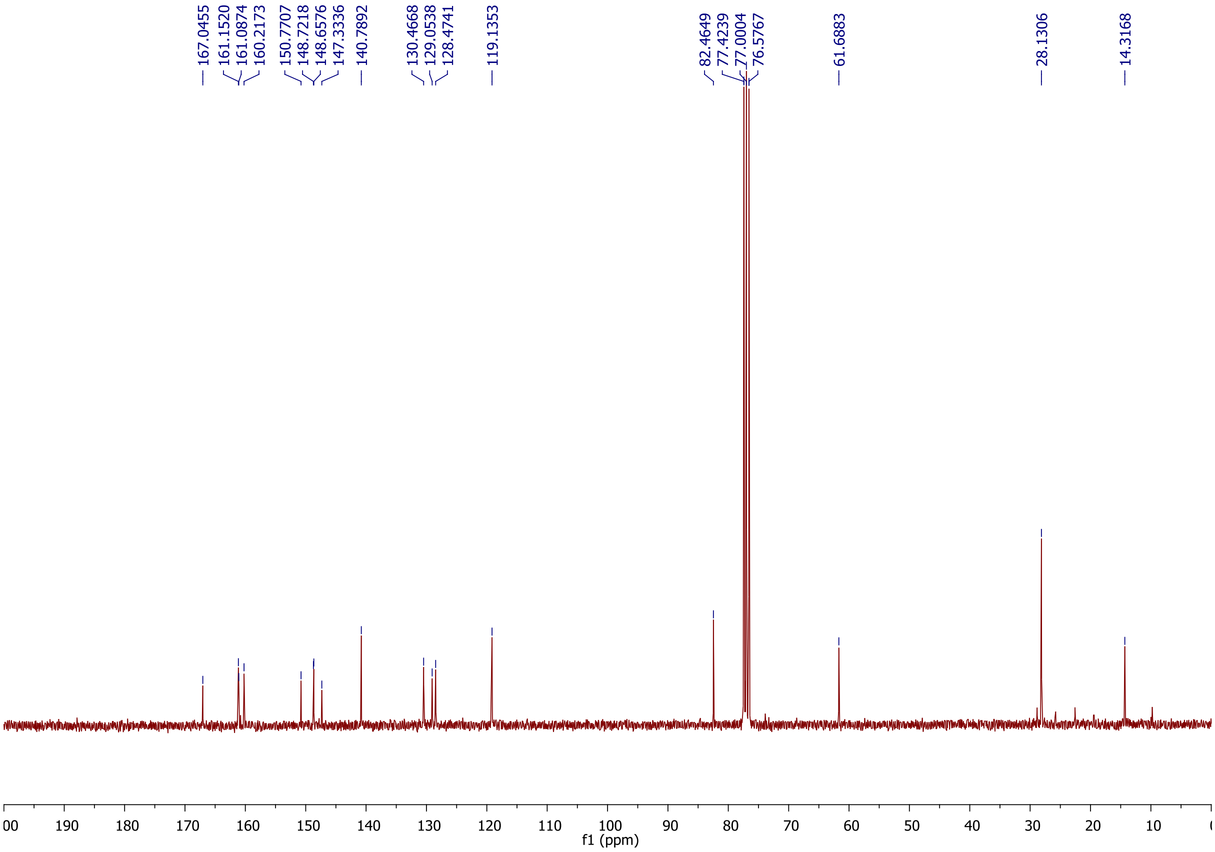

4. *tert*-Butyl 2-(2-(2-acetylthiazol-4-yl)-6-(4-(ethoxycarbonyl)thiazol-2-yl)pyridin-3-yl)thiazole-4-carboxylate (**9**).

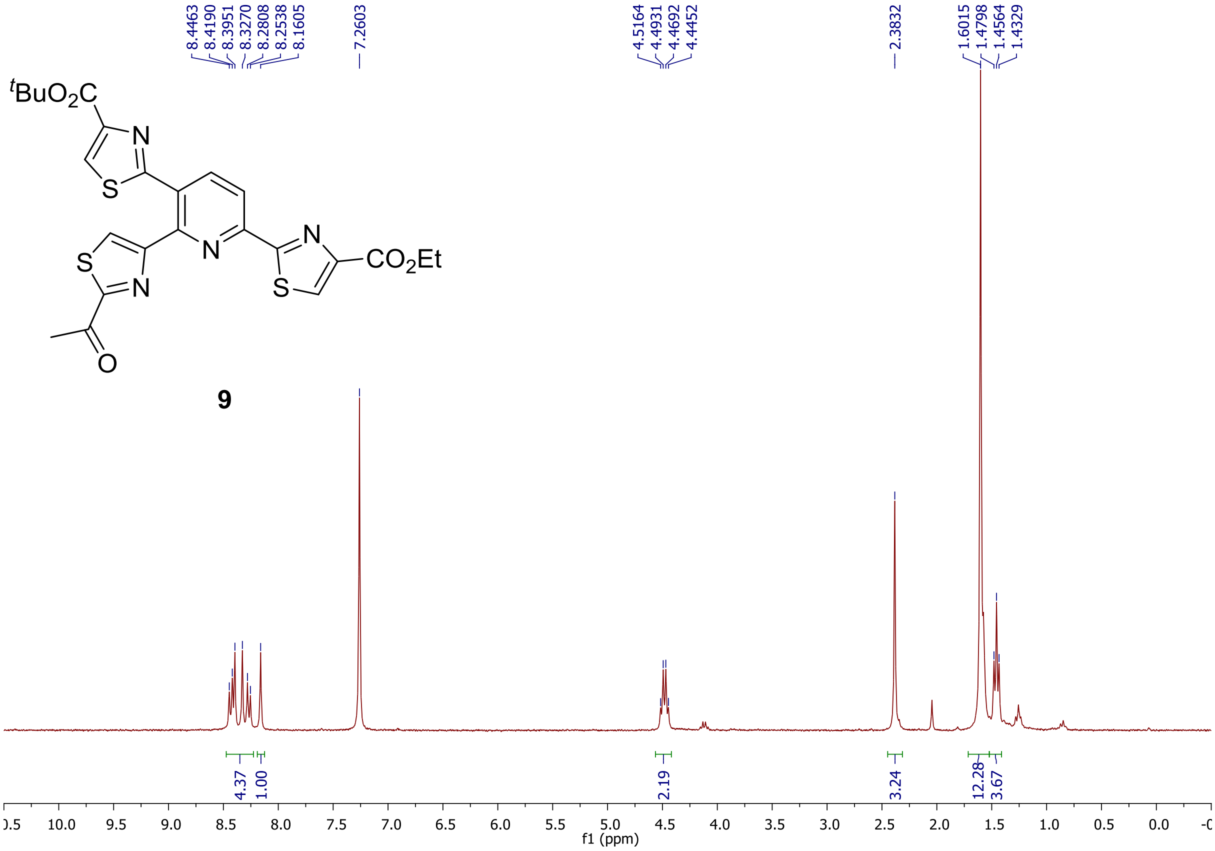

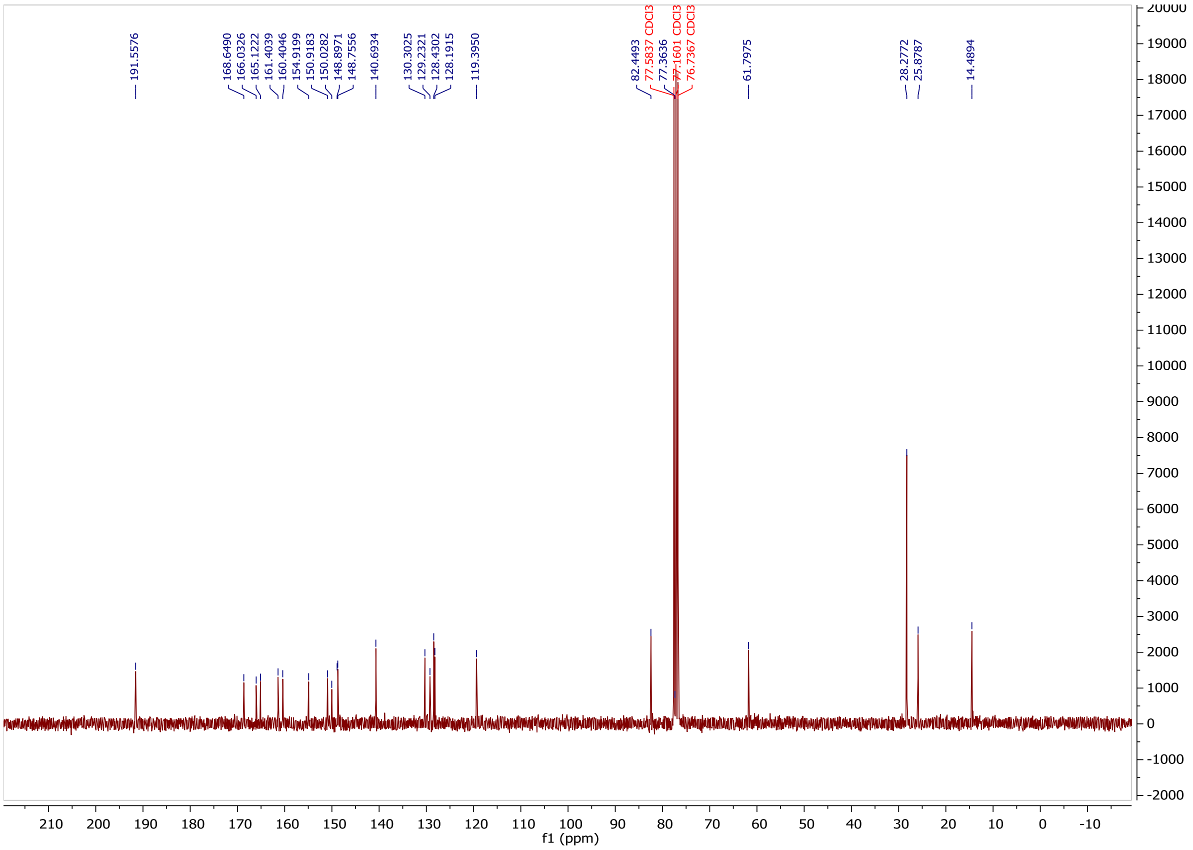

2. Synthesis of chiral thioamide 16

a) (2*S*,3*S*)-Ethyl-2-(benzyloxycarbonylamino)-3-hydroxy-3-phenylpropanoate (**13**).

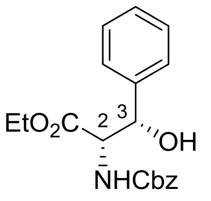

13

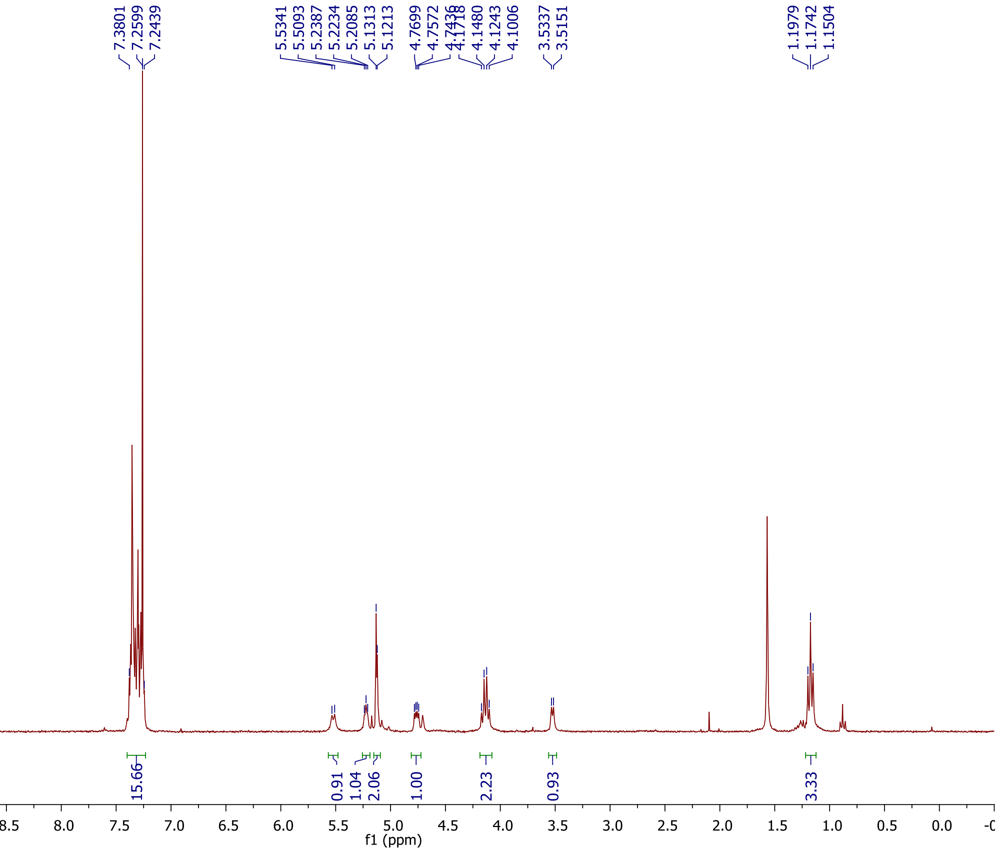

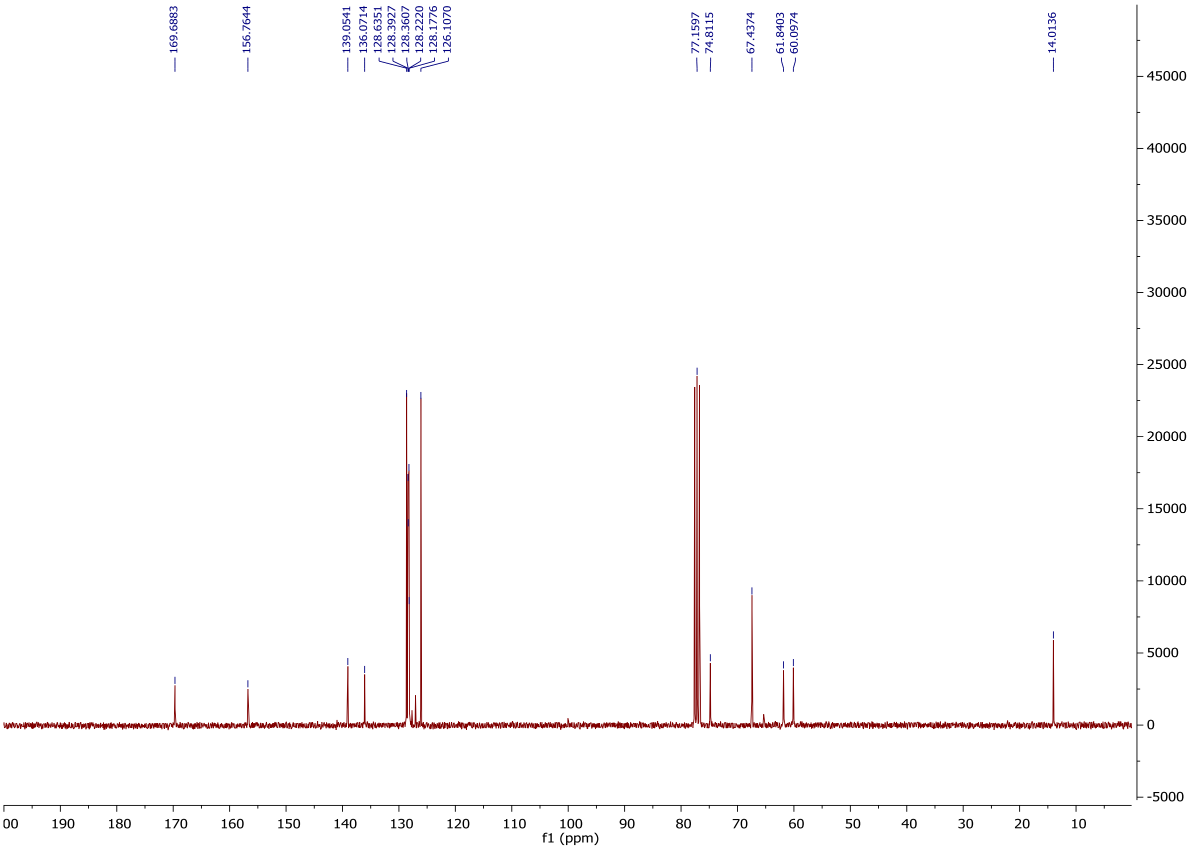

b) (2*S*,3*S*)-Ethyl 2-(benzyloxycarbonylamino)-3-(*tert*-butyldimethylsilyloxy)-3-phenylpropanoate (**14**).

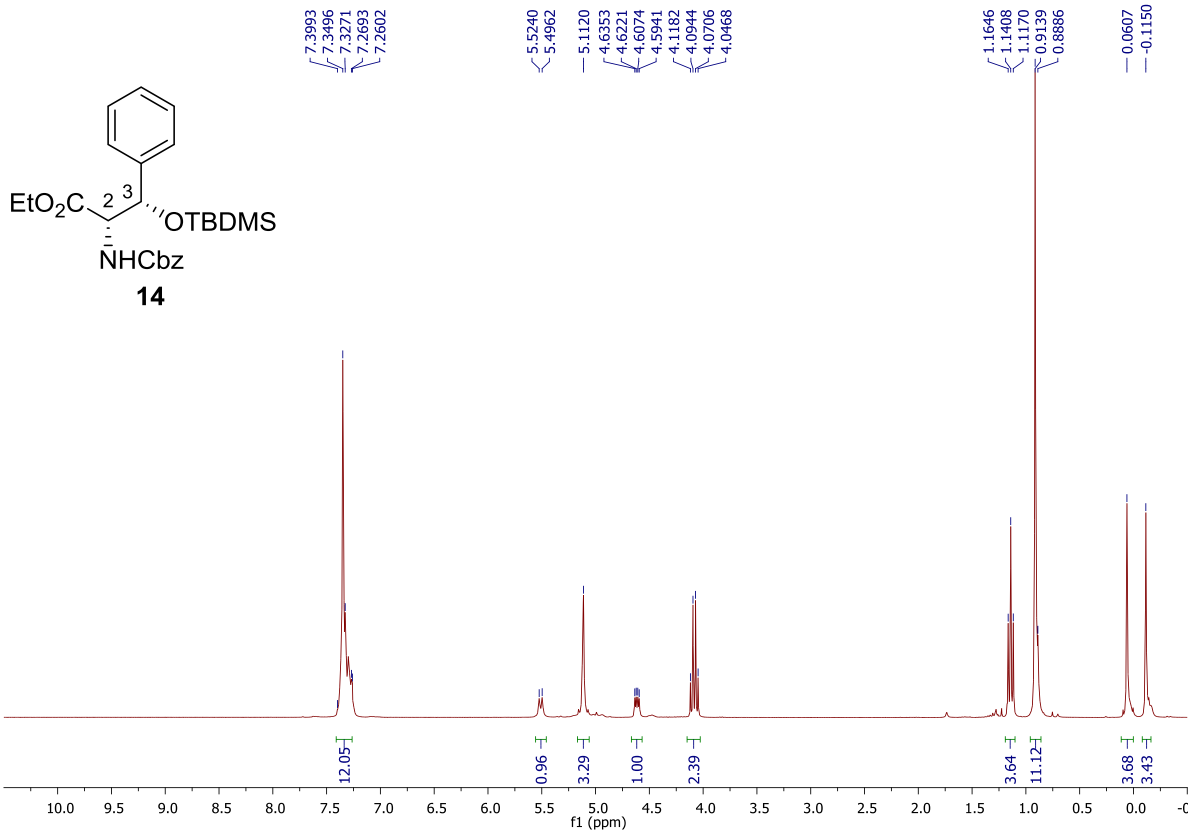

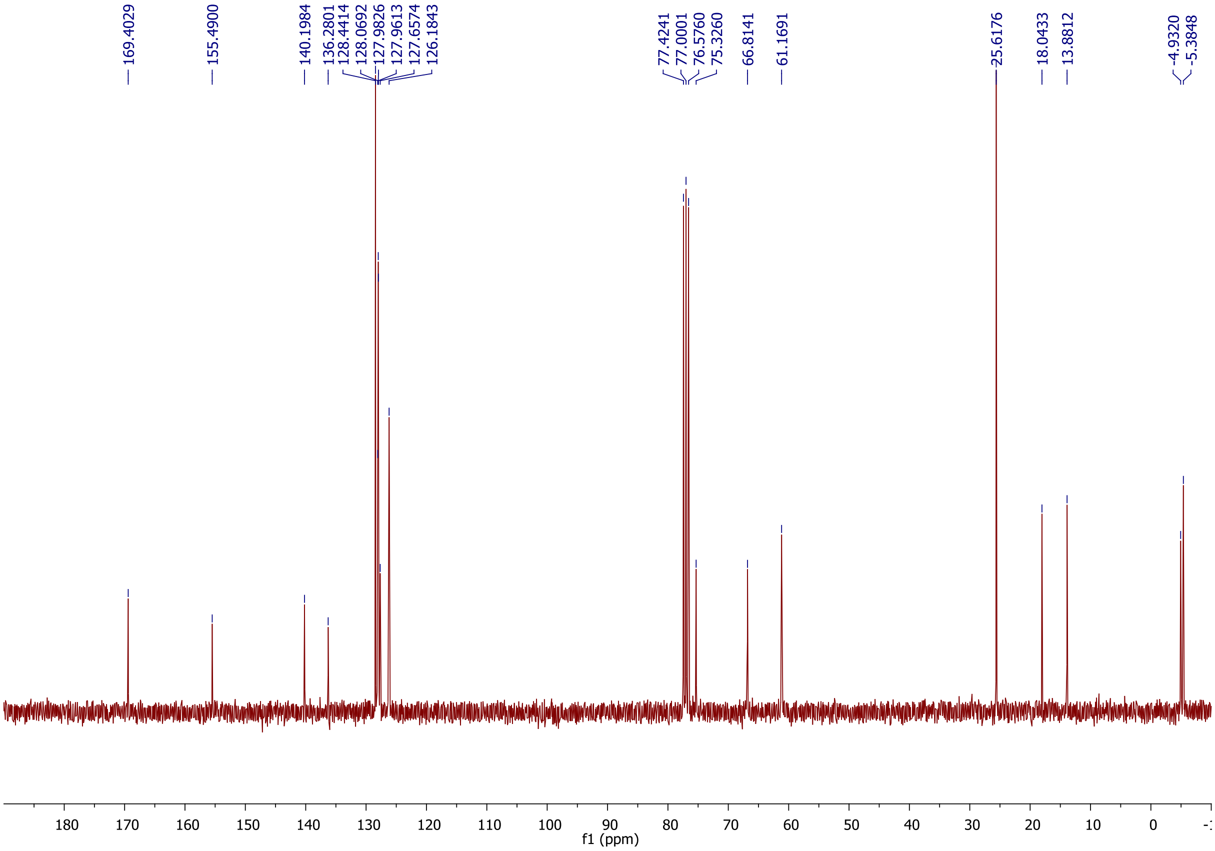

c) (2*S*,3*S*)-2-(Benzyloxycarbonylamino)-3-(*tert*-butyldimethylsilyloxy)-3-phenylpropanoic acid (**15**).

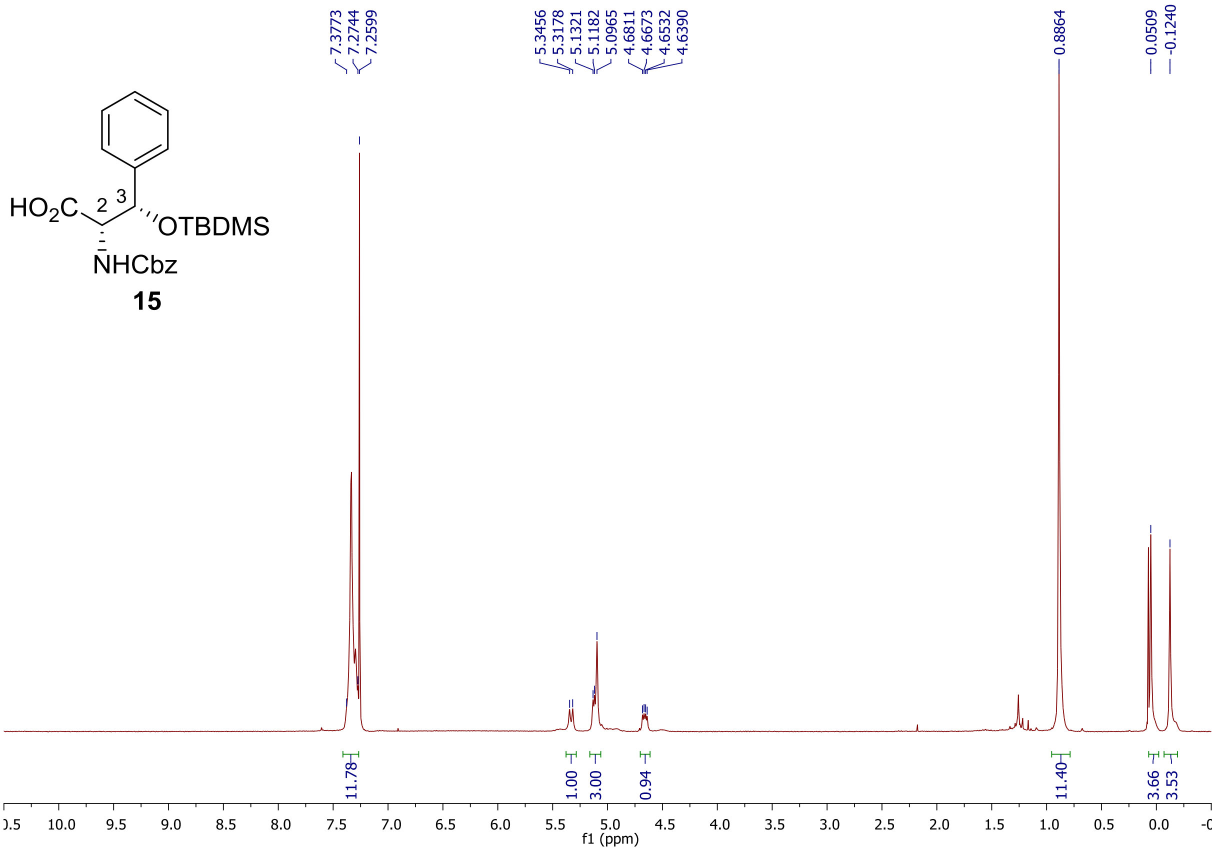

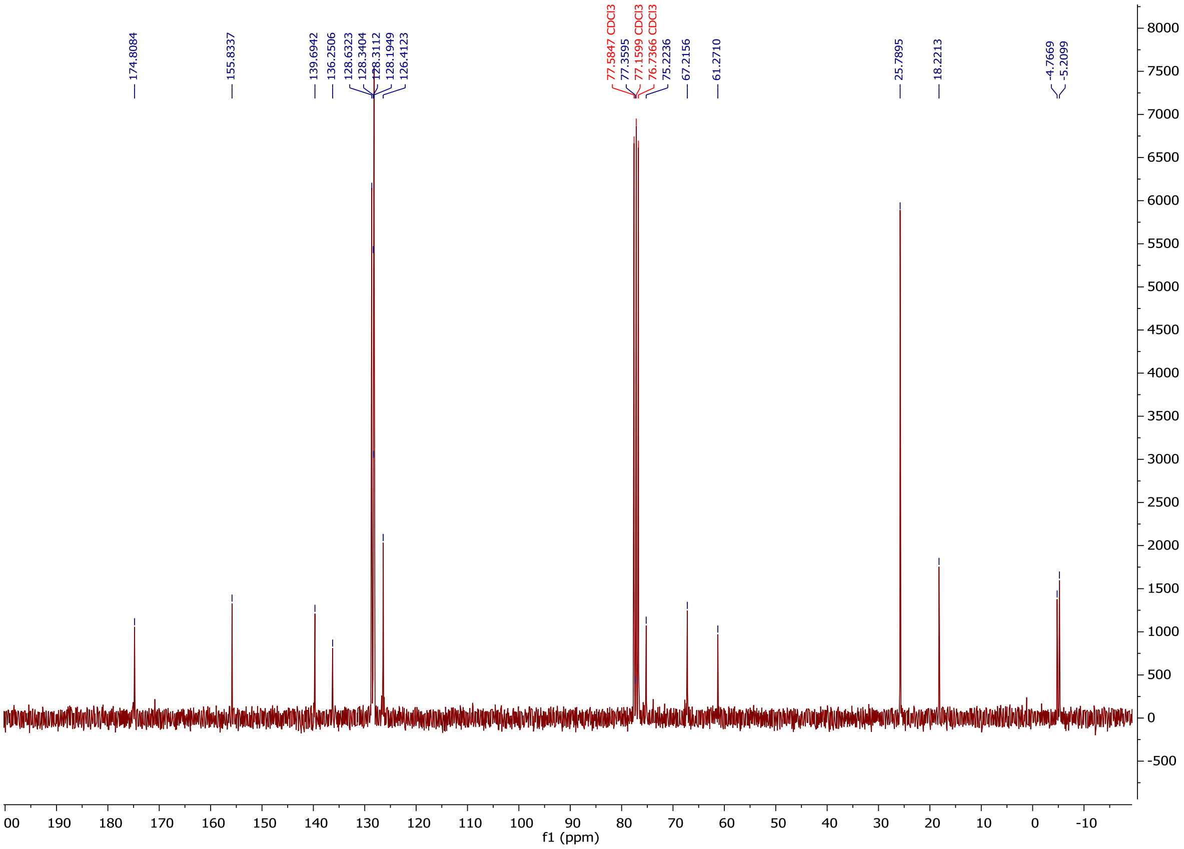

d) Benzyl (2*S*,3*S*)-1-amino-3-(*tert*-butyldimethylsilyloxy)-3-phenyl-1-thioxopropan-2-ylcarbamate (**16**).

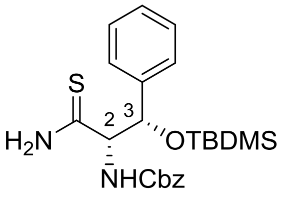

16

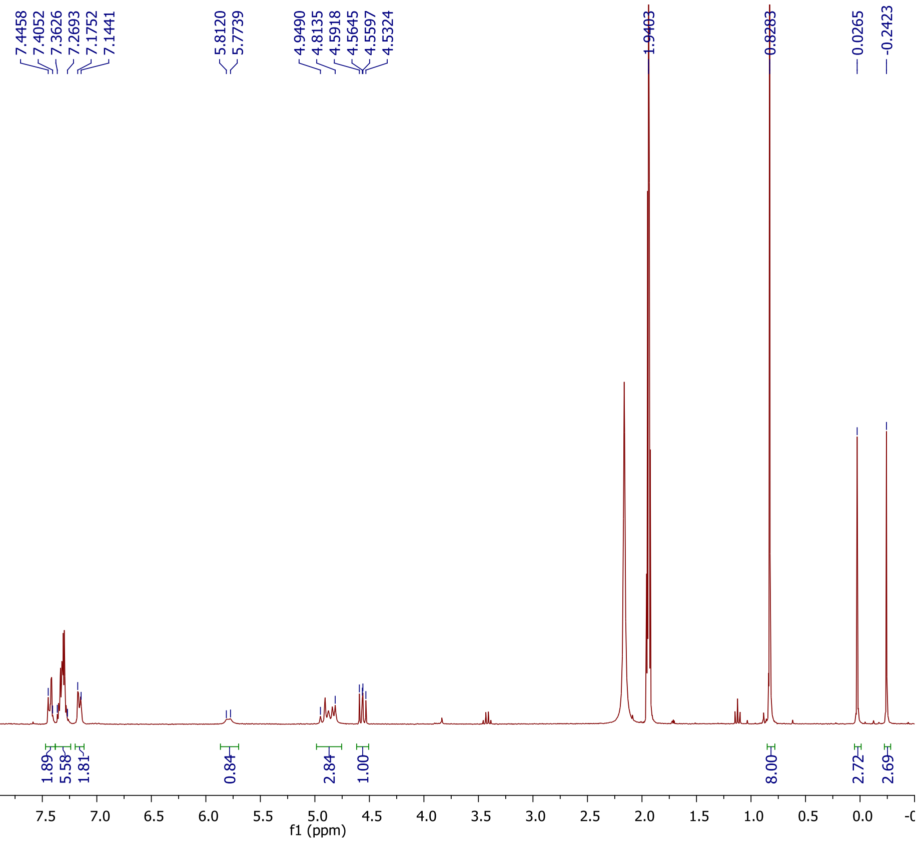

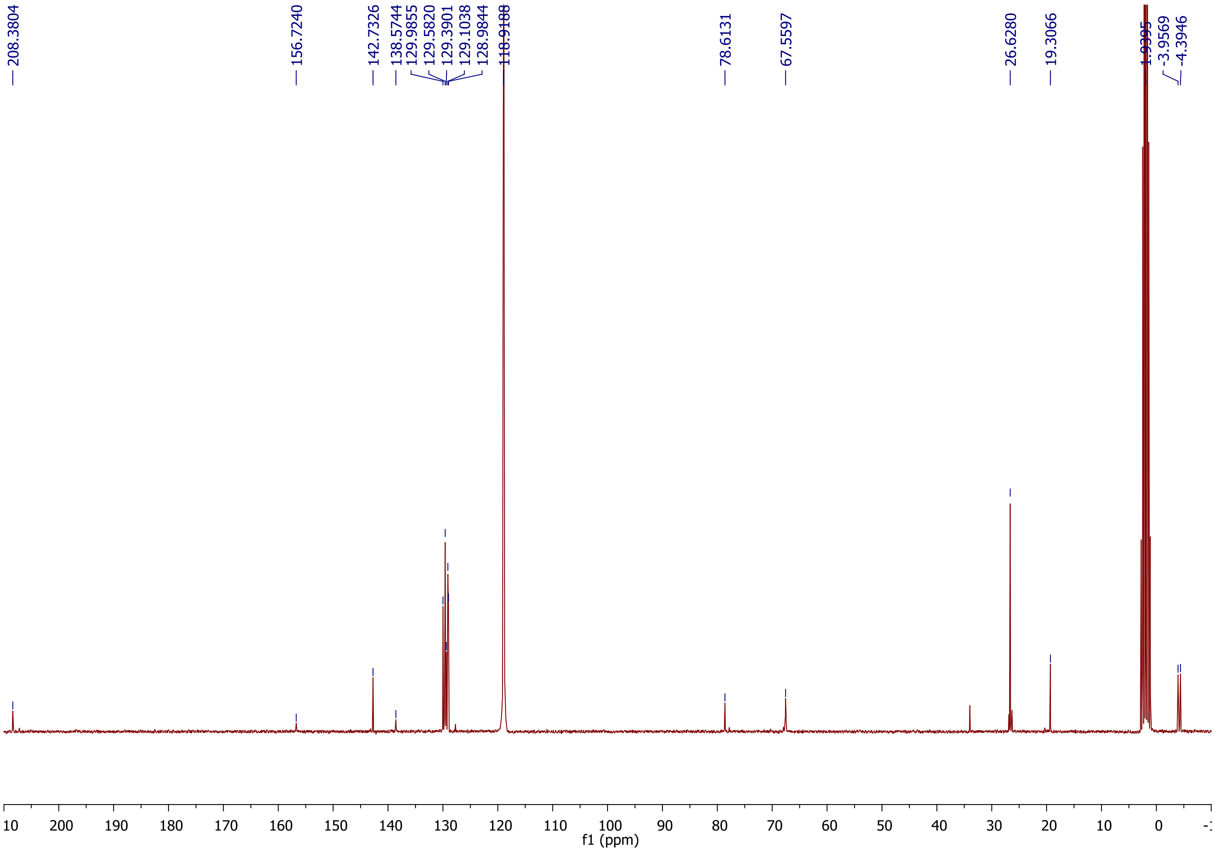

3. Synthesis Synthesis of heterocyclic core of D-series GE2270

a) *tert*-Butyl 2-(2-(2-(1-(*tert*-butyldimethylsilyloxy)vinyl)thiazol-4-yl)-6-(4-(ethoxycarbonyl)thiazol-2-yl)pyridin-3-yl)thiazole-4-carboxylate .

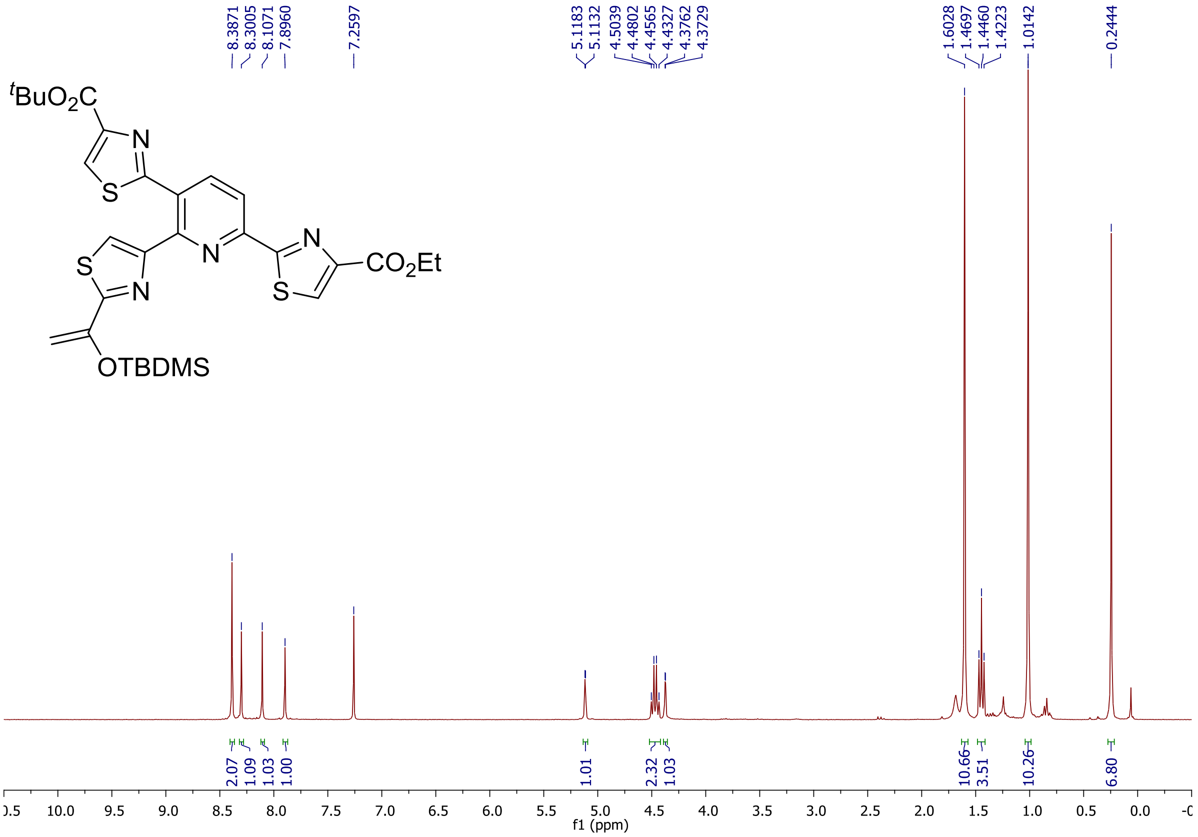

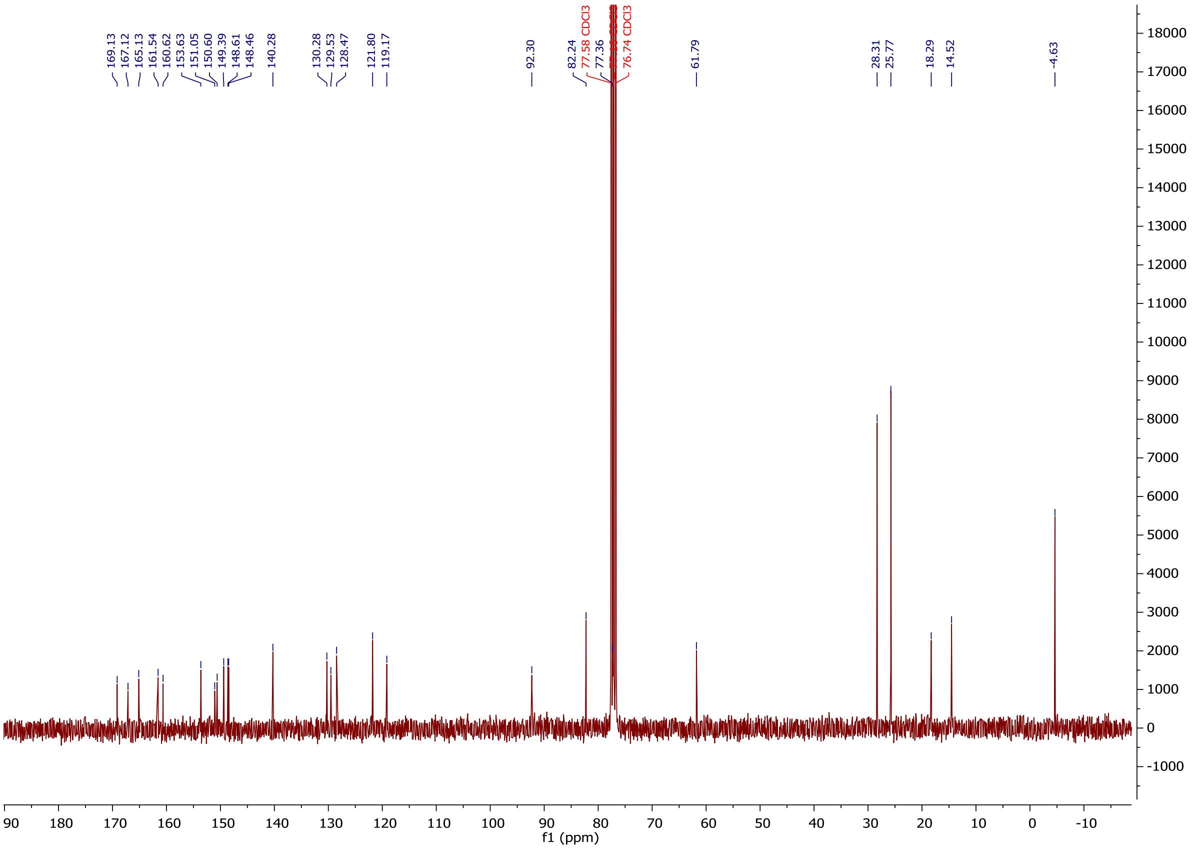

b) *tert*-Butyl 2-(2-(2-(2-bromoacetyl)thiazol-4-yl)-6-(4-(ethoxycarbonyl)thiazol-2-yl)pyridin-3-yl)thiazole-4-carboxylate (**17**).

17

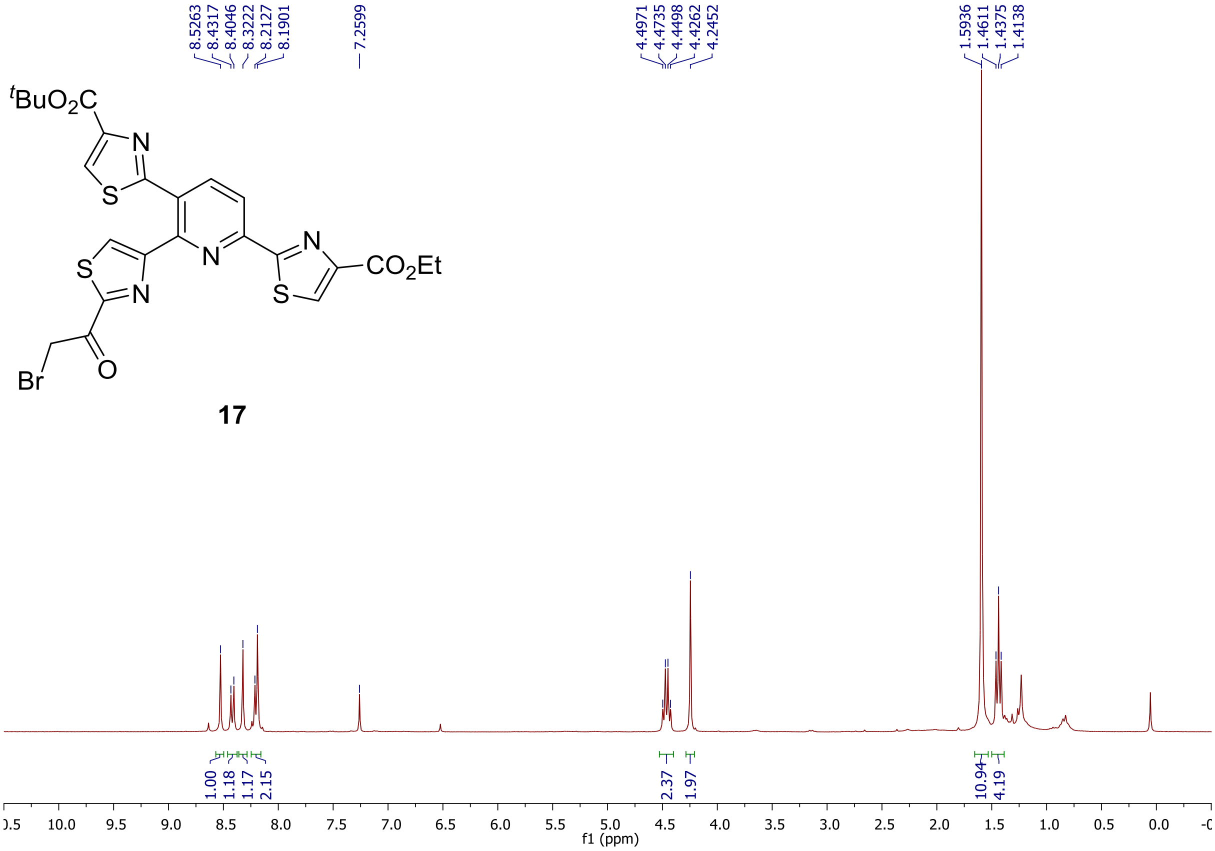

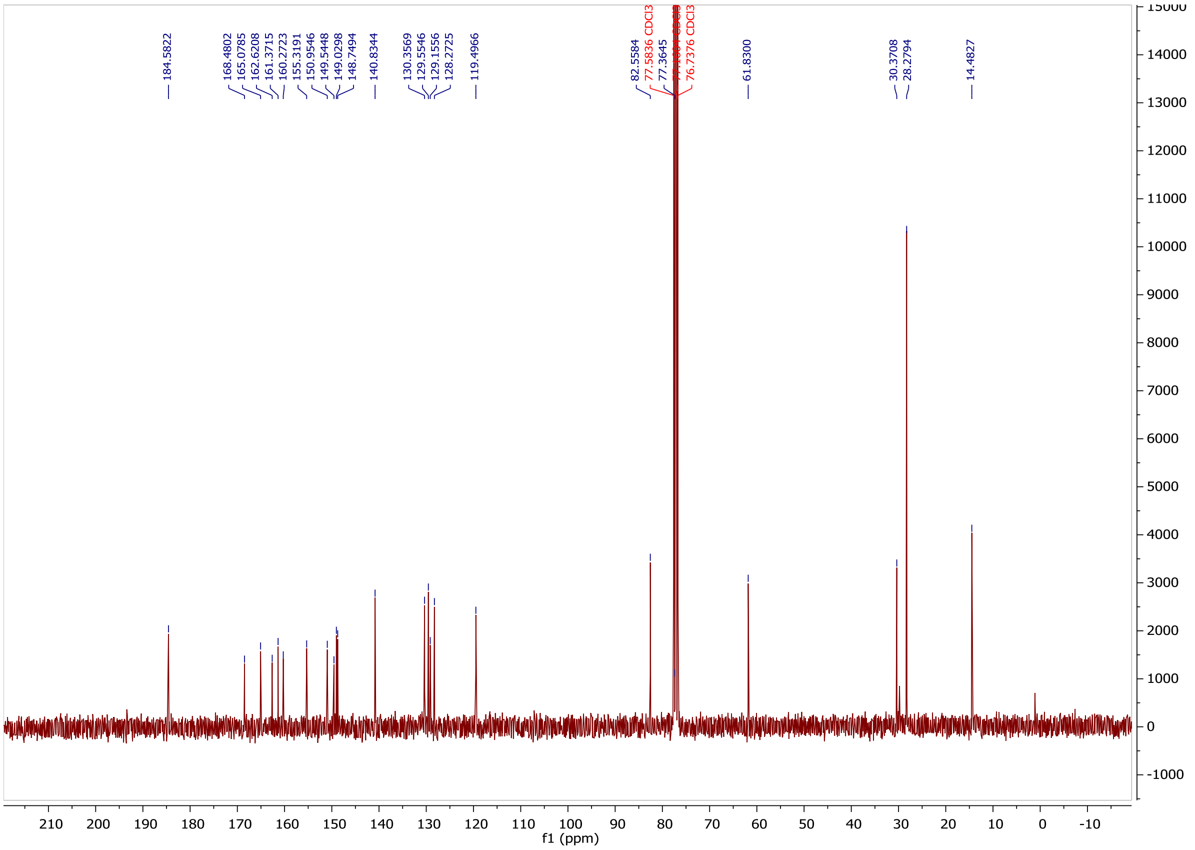

4. Synthesis of the central core GE2270A core (18).

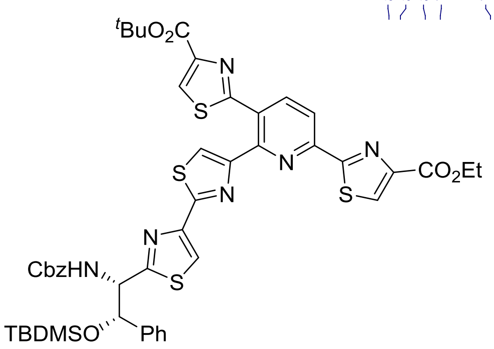

18

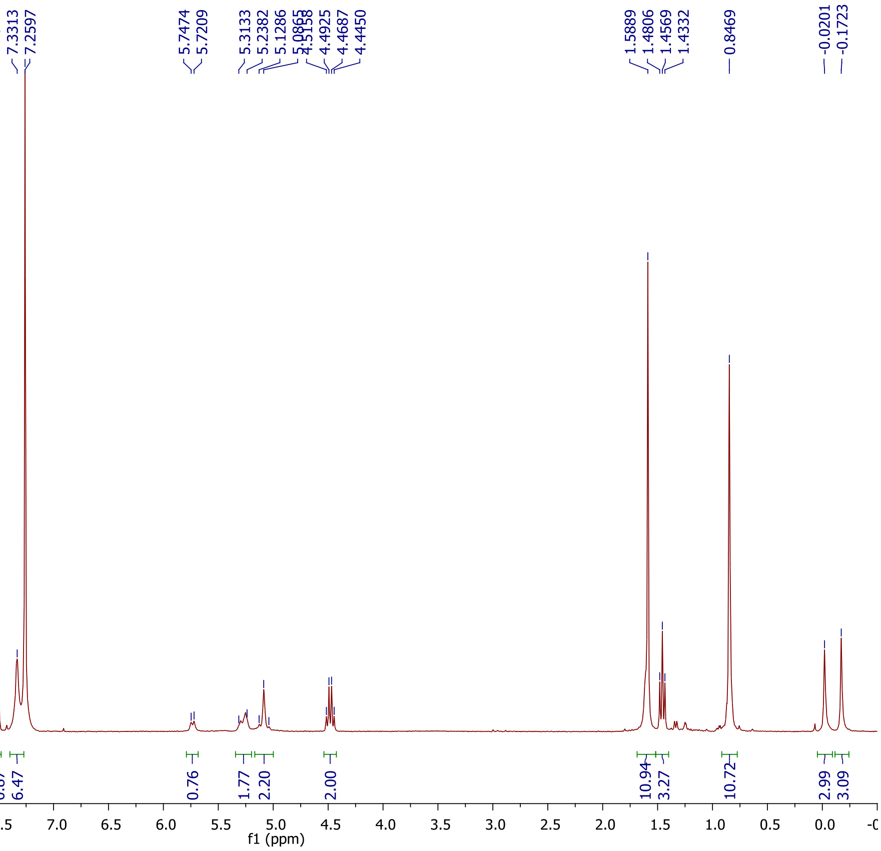

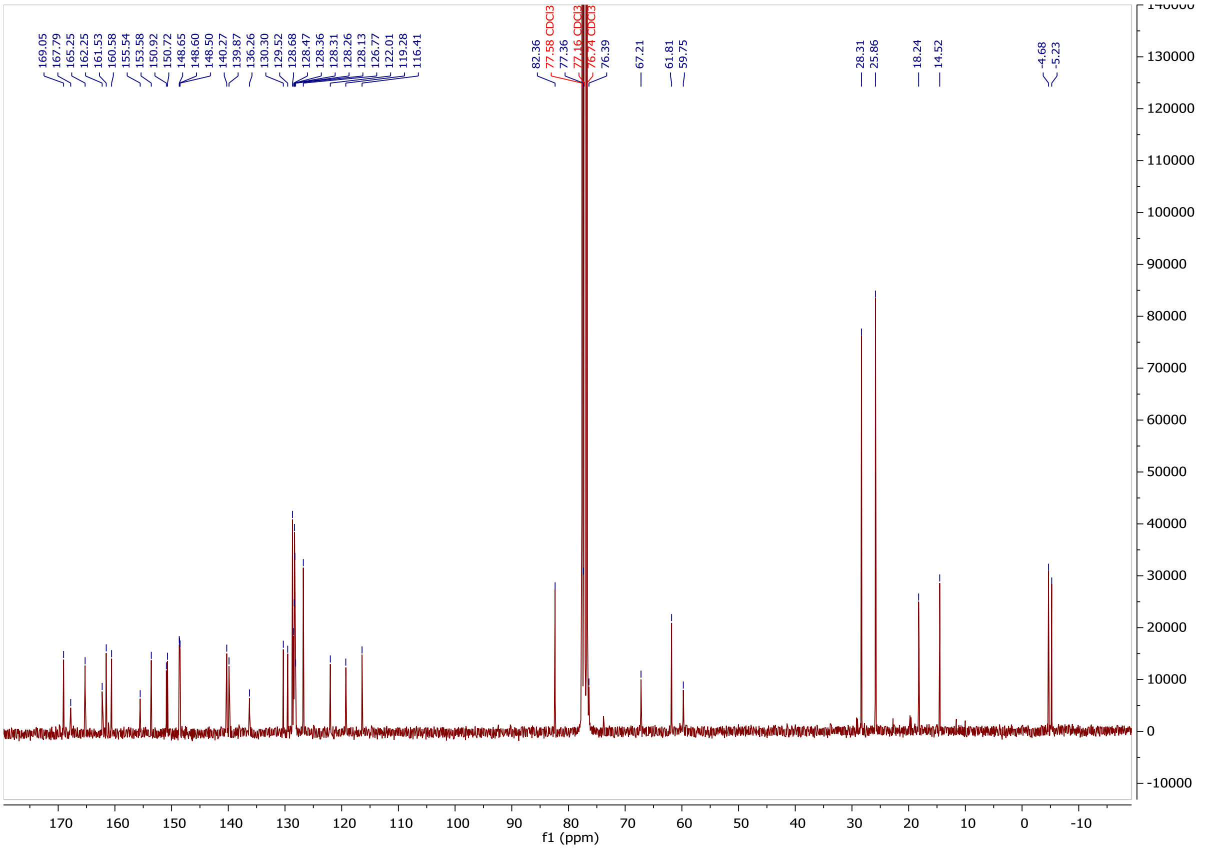

5. HPLC Chromatograms

GE2270A core

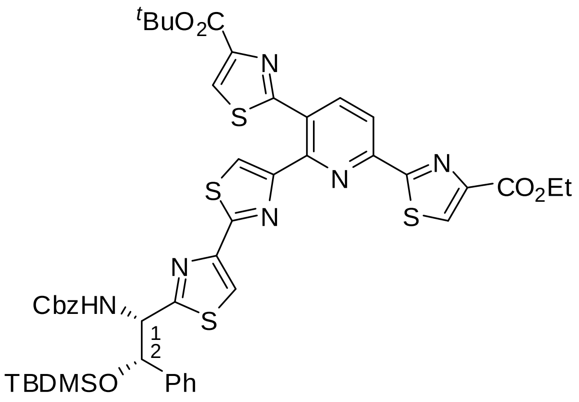

1

Racemic	99% *ee*, dr : 91:9
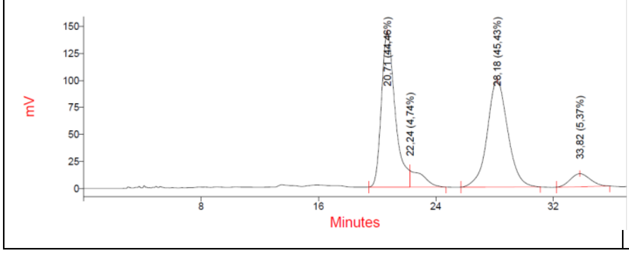	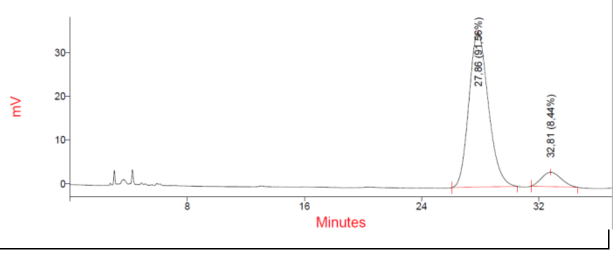
